# Global risk of selection and spread of *Plasmodium falciparum* histidine-rich protein 2 and 3 gene deletions

**DOI:** 10.1038/s41591-025-03974-3

**Published:** 2025-10-06

**Authors:** Oliver J. Watson, Thu Nguyen-Anh Tran, Robert J. Zupko, Tasmin Symons, Rebecca Thomson, Theodoor Visser, Susan Rumisha, Paulina A. Dzianach, Nicholas Hathaway, Isaac Kim, Jonathan J. Juliano, Jeffrey A. Bailey, Hannah Slater, Lucy Okell, Peter Gething, Azra Ghani, Maciej F. Boni, Jonathan B. Parr, Jane Cunningham

**Affiliations:** 1 Medical Research Council Centre for Global Infectious Disease Analysis, School of Public Health, Faculty of Medicine, Imperial College London, London, UK; 2 Center for Infectious Disease Dynamics, Pennsylvania State University, University Park, PA USA; 3 Malaria Atlas Project, Telethon Kids Institute, Perth Children’s Hospital, Nedlands, Western Australia Australia; 4 Independent Consultant, London, UK; 5 Clinton Health Access Initiative, Boston, MA USA; 6 Department of Medicine, University of Massachusetts Chan Medical School, Worcester, MA USA; 7 Center for Computational Molecular Biology, Brown University, Providence, RI USA; 8 Warren Alpert Medical School, Brown University, Providence, RI USA; 9 Department of Epidemiology, Gillings School of Global Public Health, University of North Carolina, Chapel Hill, NC USA; 10 Division of Infectious Diseases, Department of Medicine, School of Medicine, University of North Carolina at Chapel Hill, Chapel Hill, NC USA; 11 Curriculum in Genetics and Molecular Biology, School of Medicine, University of North Carolina at Chapel Hill, Chapel Hill, NC USA; 12 Department of Pathology and Laboratory Medicine, Brown University, Providence, RI USA; 13 PATH, Seattle, WA USA; 14 Faculty of Health Sciences, Curtin University, Perth, Western Australia Australia; 15 Saw Swee Hock School of Public Health, National University of Singapore, Singapore, Singapore; 16 LKC School of Medicine, Nanyang Technological University, Singapore, Singapore; 17 Nuffield Department of Medicine, University of Oxford, Oxford, UK; 18 Global Malaria Programme, World Health Organization, Geneva, Switzerland

**Keywords:** Malaria, Developing world

## Abstract

Since their first detection in 2010, *Plasmodium falciparum* malaria parasites lacking the *P. falciparum* histidine-rich protein 2 gene (*pfhrp2*) have been observed in 40 of 47 surveyed countries, as documented by the World Health Organization. These genetic deletions reduce detection by the most widely used rapid diagnostic tests, prompting three countries to switch to alternative diagnostics. However, manufacturing of alternative rapid diagnostic tests has not been scaled up and there are no World Health Organization-prequalified combination tests that use *P. falciparum* *Plasmodium* lactate dehydrogenase. The continuing spread of *pfhrp2* and/or *pfhrp3* (*pfhrp2/3*) deletions threatens malaria control, creating an emerging public health crisis. Here we use mathematical modeling informed by current *pfhrp2/3* deletion prevalence and a literature review to assess the global risk of *pfhrp2/3* deletions. We identify ten priority countries for surveillance and predict that the primary spread in Africa will move southward from the Horn of Africa through East Africa within 20 years. Despite variation in modeled timelines due to uncertainty in model parameters, four countries yet to switch rapid diagnostic tests are consistently classified as high risk under a range of model assumptions. This updated model offers refined predictions to guide *pfhrp2*/*3* policy and prioritize future surveillance efforts and innovation.

## Abstract (web summary)

A modeling approach to assess the global risk of *Plasmodium falciparum* histidine-rich protein 2 and 3 gene deletions identifies ten priority African countries for surveillance and could inform malaria control policies.

## Main

The expanded use of malaria rapid diagnostic tests (RDTs) in the last 20 years has been central to global malaria control efforts to test, treat and track all malaria infections, with 262 million RDTs distributed in 2021 by national malaria programs and 413 million sold by World Health Organization (WHO)-prequalified manufacturers^1^. The RDTs commonly deployed for diagnosis of falciparum malaria detect *Plasmodium falciparum* histidine-rich protein 2 (Pf-HRP2) and its paralog *P. falciparum* histidine-rich protein 3 (PfHRP3). However, progress against malaria is now threatened by an increase in *pfhrp2* and/or *pfhrp3* (henceforth termed *pfhrp2/3*) gene deletions resulting in false-negative RDT results. In 2014, a review was conducted that called for a harmonized approach to investigate and report *pfhrp2*/*3* gene deletions^2^. As of 2023, the WHO Malaria Threat Maps included reports of *pfhrp2/3* deletions in 40 of 47 countries surveyed worldwide^3^ and reports of *pfhrp2* deletions causing false-negative rates have been as high as 80% in the worst affected settings^4^. Once detected, there are concerns that *pfhrp2/3* deletions may be rapidly selected for, as demonstrated by observations in Eritrea and Ethiopia^4,5^. There are alternative, non-HRP2-based RDTs that target alternative antigens such as *Plasmodium* lactate dehydrogenase (pLDH). Pan-specific pLDH RDTs have not, however, been brought to scale because of their lower sensitivity compared to HRP2 and, for countries that need to both detect and distinguish between *P. falciparum* (Pf) and *P. vivax*, there are no WHO-prequalified combination tests that use Pf-pLDH instead of or in addition to HRP2 for *P. falciparum* detection. This has posed particular challenges because *pfhrp2*/*3* deletions have emerged and become dominant in several countries that require this type of combination product, for example, Eritrea, Ethiopia, Djibouti, Peru and Brazil. Most other countries continue to rely on Pf-HRP2-based RDTs as their primary malaria diagnostic tool, so emergence and spread of *pfhrp2*/*3-*deleted strains represents a growing public health crisis and poses a major obstacle to the control and eradication of *P. falciparum*.

Accurate maps of *pfhrp2/3-*deleted strains and their impact on HRP2-RDT results are needed to understand the current risk to malaria control but also to parameterize the risk of future spread. Multiple molecular surveys have been undertaken to characterize the current spread and estimated prevalence of parasites with *pfhrp2/3* deletions (genotype frequency of *pfhrp2/3* deletions). However, accurately estimating the true frequency of *pfhrp2/3* deletions, their impact on HRP2-RDT results and the risk that they pose to malaria control is challenging. One challenge is the need to harmonize estimates of *pfhrp2/3* deletions across studies with different sampling and laboratory-testing schemes, which prompted the WHO to publish methodological guidance and protocols in 2018 for studying *pfhrp2*/*3* deletions^6^. However, a review of published surveys^7^ concluded that unrepresentative surveys (sampled population not representative of the whole population, for example, sampling only from severe malaria cases or sampling HIV-positive individuals) and inconsistent study design have impaired efforts to evaluate the risk of *P. falciparum* malaria cases being misdiagnosed due to *pfhrp2/3* deletions. In addition, more recent surveys with newer laboratory techniques for detecting *pfhrp2/3* deletions have detected lower frequencies of *pfhrp2*/*3* deletions^8^ than previous surveys^9^. Second, evidence suggested that there are differences in the phenotypes associated with deleted parasites between geographical regions. For example, in the Democratic Republic of Congo (DRC), a high level of deletions (6.4%, 95% confidence interval (CI) 5.1–8.0%) was found when using asymptomatic samples from the Demographic and Health Surveys (DHS)^10^. However, a subsequent study in symptomatic patients using improved laboratory methods in the same regions found no symptomatic malaria cases with *pfhrp2* deletions^11^. In contrast, Eritrea^4,12^ and Djibouti^13,14^ are affected by a high frequency of *pfhrp2*/*3-*deleted parasites that cause symptomatic and clinically relevant infections. Furthermore, in Peru, deleted parasites emerged in settings that have never relied on HRP2-based RDTs for diagnosis, prompting speculation that deletions offer an as yet undefined selective advantage in this context beyond evasion of diagnosis^15^. These distinct phenotypes imply different immediate risks to malaria control and suggest that different evolutionary pressures are driving heterogeneous spread of *pfhrp2/3* deletions across regions^16^.

In 2017, an individual-based mathematical model of malaria transmission characterizing the drivers of selection for *pfhrp2* deletions was developed, identifying malaria transmission intensity and treatment-seeking rates for malaria infection as the two largest drivers of *pfhrp2/3* deletions^17^. However, there were insufficient data to comprehensively account for other risk factors (Extended Data Table 1), such as the impact of *pfhrp2* gene deletions on parasite fitness and the different mechanisms of selection driving the distinct spread between *pfhrp2* and *pfhrp3* deletions. In addition, limited data were available to parameterize the proportion of malaria cases diagnosed by microscopy, the level of adherence to RDT-based treatment, the crossreactivity of HRP3 epitopes to yield a positive HRP2-RDT and the incidence of nonmalarial febrile illness—all factors expected to impact the selective advantage of *pfhrp2* deletions. However, new studies and data provide improved insight into these processes. For example, HRP3 crossreactivity has been shown to be higher than previously thought, with HRP3 crossreactivity on HRP2-based RDTs sufficient to mask the effects of *pfhrp2* deletions in in vitro cultures with a high parasite density^18^. However, crossreactivity will differ between brands depending on the target epitopes of the antibodies bound to the test strips^19^ and target field data from patients in Ethiopia who are malaria symptomatic showed different performance with 46% (12 of 26) of *pfhrp2*^−^*/3*^+^ samples yielding a positive HRP2-based RDT^5^. With regard to the evolutionary mechanism driving *pfhrp2/3* selection, population genetic analyses conducted in Ethiopia concluded that *pfhrp3* deletion has arisen independently multiple times, whereas *pfhrp2* deletion likely arose more recently due to the strong positive selection resulting from an HRP2-RDT-based test-and-treat policy^5^. Understanding how strongly *pfhrp2* deletion is linked to *pfhrp3* deletion is critical—if these two deletions co-occur more than would be expected by chance (analogous to positive linkage disequilibrium (LD) but between genes on different chromosomes), the benefits for RDT performance conferred by HRP3 crossreactivity will be negated. Last, in vitro competition assays of asexual parasite fitness suggest that up to a 90% relative fitness (a 10% loss in replicative rate) may be associated with *pfhrp2* deletions^20^, although no in vivo or feeding assay studies have been conducted to assess fitness costs throughout the parasite life cycle.

In this study, we incorporated recent advances in our understanding of *pfhrp2/3* deletions and new data relevant to their spread to provide a global assessment of the risk posed by *pfhrp2/3*-deleted parasite strains. Specifically, we used a mathematical model of malaria transmission and selection of *pfhrp2* deletions to evaluate the susceptibility of each malaria-endemic region to select for *pfhrp2* deletions, once deleted parasites become established in that region. Last, we predicted the continued spread of *pfhrp2* deletions globally with a focus on sub-Saharan Africa (SSA) based on best estimates of the prevalence of *pfhrp2* deletions. The resultant maps of the risk that *pfhrp2* deletions pose can be used to guide ongoing surveillance efforts, future deployment of alternative RDTs and research to improve our understanding of the biology and threat of *pfhrp2/3* deletions.

## Results

### Fitness and crossreactivity estimation for *pfhrp2* and *pfhrp3*

**Fig. 1:**
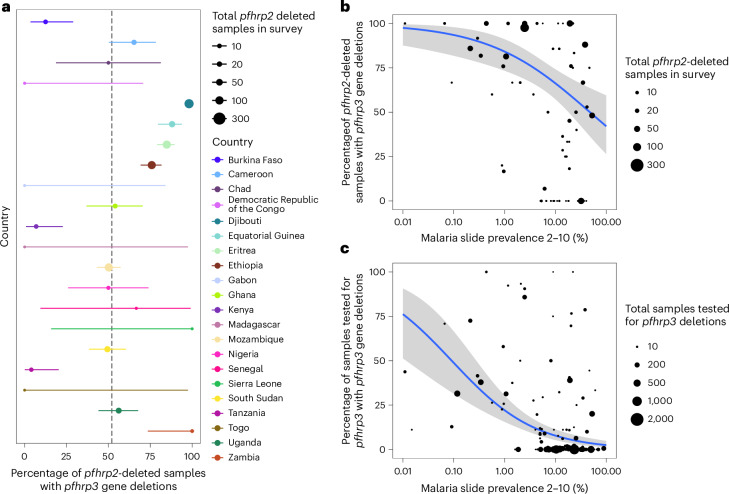
Distribution and independence of *pfhrp2/3* deletions in Africa among surveys of symptomatic individuals collated from the WHO Malaria Threat Maps database. **a**, Percentage of *pfhrp2-*deleted samples together with *pfhrp3* deletions. The mean and 95% CI are shown with points and ranges, with the vertical dashed line indicating the continent-wide estimate based on a beta-binomial model. The sample size per country is: Burkina Faso (32), Cameroon (49), DRC (3), Djibouti (272), Eritrea (198), Ethiopia (195), Gabon (2), Ghana (37), Equatorial Guinea (92), Kenya (29), Madagascar (1), Mozambique (201), Nigeria (18), Senegal (3), Sierra Leone (2), South Sudan (85), Chad (10), Togo (1), Tanzania (25), Uganda (73) and Zambia (12). **b**, Relationship between the percentage of *pfhrp2-*deleted samples with *pfhrp3* deletions and malaria slide prevalence in 2–10 year olds based on the Malaria Atlas Project estimates. **c**, Relationship between the percentage of samples with *pfhrp3* deletions and malaria prevalence. In all plots, the point size represents the number of samples from each survey used to derive estimates. In **b** and **c**, an overdispersed binomial regression model fit (blue) shows the mean relationship, with the 95% CI of the regression fit shown with shaded bands. Data is the from WHO Malaria Threat Maps database^3^.

To estimate the risk posed by *pfhrp2/3* deletions, we first estimated the probability of *pfhrp3* deletions co-occurring with *pfhrp2* deletions and the fitness costs associated with *pfhrp2* deletions based on data in the Horn of Africa. Using survey data reporting the prevalence of *pfhrp2* and *pfhrp3* deletions from the WHO Malaria Threat Maps database^3^, we estimated that, globally, 61.7% (95% CI 55.3–67.8%) of *pfhrp2*-deleted samples also had *pfhrp3* gene deletions. The distribution across studies of the percentage of *pfhrp2-*deleted samples with *pfhrp3* gene deletions was highly overdispersed, with clear differences between countries. Focusing on studies conducted in Africa (Fig. 1), we estimated that 52.1% (95% CI 42.9–60.9%) of *pfhrp2*-deleted samples also had *pfhrp3* gene deletions (Fig. 1a). We observed significant nonrandom association (*χ*^2^ = 1,747.9, degrees of freedom = 1, *P* < 2.2 × 10^−16^) based on the observed counts of *pfhrp2*- and *pfhrp3*-deleted parasites (Table 1). A further strong indication of the positive linkage between *pfhrp2* and *pfhrp3* deletions was observed based on the normalized coefficient of LD (= 0.557).

**Table 1:** Frequency of *pfhrp2/3* deletions in Africa

	*pfhrp3* deleted	*pfhrp3* wild-type	Total
***pfhrp2*** **deleted**	595	851	1,446
***pfhrp2*** **wild-type**	392	7,736	8,128
**Total**	987	8,587	9,574

The table shows the total number of samples categorized by *pfhrp2/3* gene deletion from studies available in the WHO Malaria Threat Maps database^3^.

We found a significant negative relationship between malaria prevalence and *pfhrp3* gene deletion prevalence among *pfhrp2*-deleted samples with a log(odds ratio) (log(OR)) of −0.3017 (95% CI −0.3340 to −0.2695, *P* < 2.2 × 10^−16^) (Supplementary Table 1), with surveys conducted in regions with higher malaria prevalence less likely to observe *pfhrp2-*deleted samples among samples with *pfhrp3* deletions (Fig. 1b). We also observed significantly lower frequencies of *pfhrp3* deletions in surveys conducted in regions with higher malaria prevalence (Fig. 1c). A different relationship between *pfhrp2/3* independence and malaria prevalence was observed on the other continents; studies in Asia showed insignificant associations between *pfhrp3*-deletion frequency and malaria prevalence (Supplementary Fig. 1).

Using longitudinal data on *pfhrp2/3* deletions from Eritrea and Ethiopia, we fit our malaria transmission model to jointly infer parameter values for both the fitness costs of *pfhrp2* deletions and the crossreactivity of HRP3 epitopes. We estimated relative fitness of 96.4% (95% CI 95.3–97.3%) for *pfhrp2* deletions (that is, the relative contribution of deleted parasites to onward infections each day = 96.4% of that from wild-type parasites). From the results of the same model fitting, we estimated the probability that an infection due to only *pfhrp2*-deleted parasites would still produce a positive HRP2-based RDT (HRP3 crossreactivity producing a positive test outcome) of 25.0% (95% CI 14.0–41.0%) (Supplementary Fig. 2).

### Modeling the impact of drivers of selection for *pfhrp2* deletions

To fully evaluate the risk of selection for *pfhrp2/3* deletions, we conducted a literature review to identify and extract estimates for additional known drivers of *pfhrp2/3* selection (Extended Data Table 1). We found parameter estimates for most of the risk factors identified; however, sources from reported WHO national data or the academic literature failed to identify suitable estimates for all malaria-endemic countries. To address this, we identified previous efforts by other groups that produced modeled parameter estimates at either the national or the first administrative unit, notably the Commodities Forecast Dashboard by the Malaria Atlas Project^21^. When compared against our literature review, we found broad agreement in the data sources identified (Supplementary Information), which resulted in similar estimates as produced by the Malaria Atlas Project for modeling trends in malaria commodities^22^. However, we identified a number of outliers, totaling <0.5% of all parameters collected. These included outliers that reflected gaps in nationally reported data, for example, zero reported cases to the WHO of malaria tested by RDT and edge cases, such as ~100% of individuals with care-seeking malaria infections, who are not tested, receiving treatment. In response, outliers were identified and multiple imputations using random Forest plots were used to correct outliers based on the other collected covariates, yielding global maps of each parameter (Supplementary Figs. 3–6).

Using our model of malaria transmission, we conducted 43,740 simulations across the full range of parameters identified for each country. We trained an ensemble machine learning model to accurately predict selection coefficients based on these simulations (Supplementary Fig. 7). Based on the partial dependence of the ensemble statistical model, we identified malaria prevalence as the most important determinant of the selection of *pfhrp2* deletions (Supplementary Fig. 8), with selection of *pfhrp2* deletions notably increasing at malaria prevalence <20% based on microscopy slide prevalence in 2–10 year olds (Supplementary Fig. 8a). Treatment cascade parameters (nonadherence to RDT test outcomes, use of non-HRP2-based RDTs for testing and the HRP3 crossreactivity) had similar inferred effect sizes on the selection of *pfhrp2* deletion (Supplementary Fig. 8c–f), reflecting their similar role in altering the probability that an individual is treated based only on the outcome of an HRP2-based RDT.

### Mapping the risk posed by *pfhrp2* deletions

**Fig. 2:**
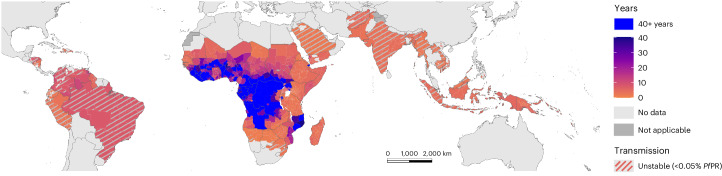
Global distribution of predicted times in years for the percentage of clinically relevant infections misdiagnosed due to *pfhrp2/3* gene deletions to increase from 1% to 5%. Regions estimated not to reach 5% within 40 years are shown in blue. Regions with very low, unstable malaria transmission (defined as <0.05% malaria slide prevalence in 2–10 year olds) are shown with diagonal gray lines (see Supplementary Fig. 9 for focus on Africa). *Pf*PR, *P. falciparum* prevalence.

We quantified the risk posed by *pfhrp2/3* deletions using two approaches: the ‘innate risk’ and ‘prospective risk’. First, we estimated the ‘innate risk’, defined as the time taken for the percentage of clinical cases to be misdiagnosed by Pf-HRP2-based RDTs to increase from 1% to 5% (WHO threshold to switch to non-HRP2-based RDTs) in each region, if using only HRP2-based RDTs. The innate risk captures the innate susceptibility of each region to select for deletions once established. We predicted that 73 of 106 countries modeled would have at least 1 first administrative unit reach the 5% threshold within 20 years (Fig. 2). We predicted that most of the highest-risk regions are very low transmission regions (<0.05% malaria prevalence); however, evolutionary trajectories in these settings are highly uncertain. The very low malaria prevalence, and consequently the small effective population size, is predicted to increase the stochasticity in the dynamics of *pfhrp2* deletions—similar to classic findings of the relationship between genetic drift and selection^23^. Consequently, there is also the increased chance that deleted strains will stochastically fade out due to small malaria population size, rather than increasing despite conditions being favorable for the selective advantage conferred by *pfhrp2* deletions to be realized. Conversely, we predicted a low risk of *pfhrp2* deletions in the highest malaria prevalence regions in Central and Western Africa, with estimated times to reach the 5% threshold in excess of 40 years.

**Fig. 3:**
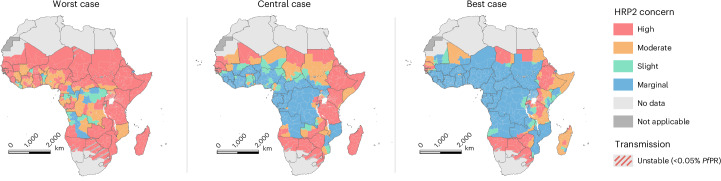
Innate risk score for the concern caused by *pfhrp2* deletions in Africa. High (red), moderate (yellow) and slight (teal) risk representing >5% of clinically relevant infections misdiagnosed due to *pfhrp2/3* gene deletions in <6, 12 and 20 years, respectively, and marginal risk (blue) representing <5% in 20 years. Uncertainty in model parameters for each region impacts the risk scores, with the worst-case (right), central-case (middle) and best-case (right) scenarios (based on the uncertainty in the range of parameters explored) shown. Regions with very low, unstable malaria transmission (defined as <0.05% slide prevalence in 2–10 year olds (*Pf*PR)) are shown with diagonal gray lines (see Supplementary Fig. 10 for global risk scores).

Focusing on countries with >0.05% estimated malaria slide prevalence in 2020, we identified 20 countries in which most of the first administrative units were classified as high innate risk (reaching the 5% threshold within 6 years) (Table 2). All but three countries (Solomon Islands, Papua New Guinea and Guyana) are in Africa, with most of these countries in Africa representing those in which *pfhrp2/3* deletions have already been identified (for example, Djibouti, Eritrea, Ethiopia and Gambia). Notably, a few regions in Djibouti are predicted to have a marginal risk, which reflects the recent increases in malaria transmission and contrasts with the previously lower malaria prevalence in these regions, which would have increased selection for *pfhrp2/3* deletions. However, we found a large range in assigned risk scores when we compared risk scores across the range of parameter uncertainties for each region (Fig. 3). Most of the uncertainty in selection speed for *pfhrp2* deletions is due to wide uncertainties in malaria prevalence for each first administrative unit. For example, malaria prevalence estimates in Yobe, Nigeria for 2020 range between 10% and 40%, which corresponds to an absolute change in selection coefficient of 0.3 (that is, an absolute increase of 30% in the annual proportional change in *pfhrp2* deletions). This change in predicted selection coefficients would result in a change in regional classification from marginal concern (1–5% in >20 years) to high concern (1–5% in <6 years). Despite this uncertainty, we identified a number of regions that are consistently classified as high concern across the range of parameter uncertainties, such as in Eritrea, Ethiopia, Zambia and Tanzania, and a number of regions in Central and West Africa that are consistently classified as marginal risk (1–5% in >20 years).

**Table 2:** High-risk countries by risk score

Country	Percentage of first administrative units with high innate risk
Comoros	100.0
Eritrea	100.0
Ethiopia	100.0
Gambia	100.0
Guyana	100.0
Madagascar	100.0
Namibia	100.0
Papua New Guinea	100.0
Rwanda	100.0
Senegal	100.0
Tanzania	100.0
Zimbabwe	100.0
Solomon Islands	100.0
Kenya	93.6
Guinea-Bissau	88.9
Yemen	84.2
Guyana	80.0
Mauritania	66.7
Djibouti	60.0
Somalia	50.0
Zambia	50.0

Country	Percentage of first administrative regions with high prospective risk
Djibouti	100.0
Eritrea	100.0
Ethiopia	100.0
Senegal	100.0
South Sudan	100.0
Sudan	100.0
Kenya	95.7
Ghana	90.0
Equatorial Guinea	85.7
Zambia	60.0

The percentage of first administrative units classified as high innate or prospective risk (>5% of clinically relevant infections misdiagnosed due to *pfhrp2/3* gene deletions in <6 years, given a starting frequency of *pfhrp2* deletions of 1%) is shown. Only countries in which ≥50% regions are classified as high risk are shown. The data presented are derived from the mathematical modeling detailed in Methods.

**Fig. 4:**
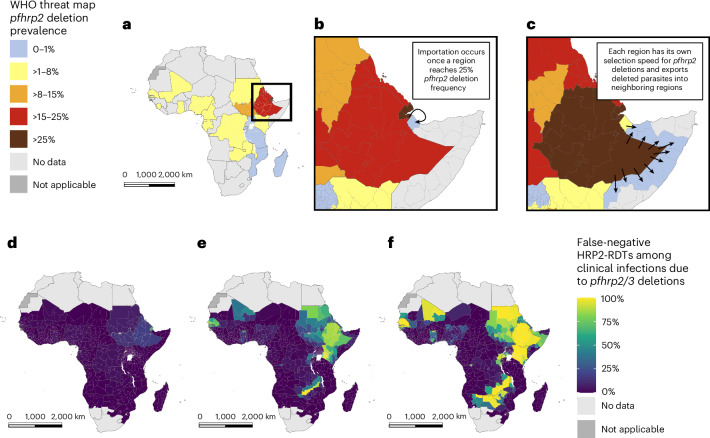
Prospective risk scores for *pfhrp2* deletions in Africa. **a**, The prospective risk score modeling continued spread of deletions based on current best estimates of the prevalence of *pfhrp2* deletions as collated in the WHO Malaria Threat Maps database (2023)^3^. **b**,**c**, In this model, assumptions that deletions are imported into a region from a neighboring region once they have reached a prevalence of 25% (**b**, 2024) and selection of deletions in a region determined by that region’s transmission intensity and treatment-related parameters (**c**, 2025). **d–f**, The predicted spread of false-negative RDTs due to *pfhrp2/3* deletions in Africa over the next 20 years: 2023 (**d**); 2033 (**e**); 2043 (**f**). The color bar shows the percentage of clinically relevant infections misdiagnosed due to *pfhrp2/3* gene deletions (Supplementary Video 1).

Although the innate risk captures the underlying selection dynamics, it does not incorporate data on the current distribution of *pfhrp2/3* deletions in Africa. Consequently, we also estimated the ‘prospective risk’, which is calculated using simulations of the continued spread of *pfhrp2* deletions in Africa, based on current estimates of *pfhrp2/3* deletions from the WHO Malaria Threat Maps^3^ and assuming that countries maintain their existing RDT procurement and usage patterns. In Africa, we predicted that 28 of the 49 countries modeled have at least one first administrative unit predicted to reach the 5% threshold or have already reached the 5% threshold within 20 years (Fig. 4). If HRP2-based RDTs remain the mainstay of malaria case management, we predicted that the major route for *pfhrp2* deletions is to spread south out from the current hotspot in the Horn of Africa, moving through East Africa over the next 20 years. In addition, deletions identified in Western Africa are predicted to increase, especially in Senegal and Mali. Prospective risk scores classified fewer regions as high risk than innate risk scores (Supplementary Fig. 11). Across both risk scores, however, a number of countries are predicted to be identified as being high risk in the majority (>50% of first administrative units; Table 2), including Djibouti, Eritrea, Ethiopia, Senegal, Zambia and Kenya. Similar to the innate rsk score, there is considerable uncertainty in the modeled timelines for the spread of deletions. Interactive risk maps for each parameter scenario are available at https://worldhealthorg.shinyapps.io/DeletionRiskExplorer (Supplementary Fig. 12).

## Discussion

In this study, we modeled the global risk of selection and spread of *pfhrp2* deletions and confirmed the threat they pose to malaria control efforts in Africa if case management continues to rely on HRP2-based diagnosis. Incorporating the most recent understanding of deletions and the best estimates of key model parameters, we found that malaria prevalence was the most important driver of deletions globally. However, uncertainty in malaria prevalence data, further exacerbated by the pandemic-induced delay in key data sources such as the DHS, limits confidence in regional risk estimates. In response, we investigated a range of scenarios and uncertainties to identify countries and regions at highest risk from deletions across the range of scenarios explored. Globally, most malaria-endemic areas, and especially those with very low prevalence, are predicted to select for deletions rapidly. In Africa, this includes regions in the Horn of Africa, East Africa and a few countries in West Africa, such as Senegal and Mali.

Our findings contrasted with earlier *pfhrp2* deletion risk maps and timelines^17^ in several notable ways. First, our approach focused on a different outcome measure, namely the proportion of clinically relevant malaria cases misdiagnosed due to gene deletions, consistent with current WHO policy guidance^24^. Second, we incorporated the best available data on current deletion prevalence to evaluate how deletions may spread between regions and focused only on surveys of symptomatic patients to ensure that our estimates of *pfhrp2* and *pfhrp3* deletions align with the criteria for the WHO 5% threshold. Third, we produced an interactive tool for decision-makers to explore the risk maps for each parameter scenario and understand how each parameter impacts the selection of *pfhrp2* deletions. However, despite incorporating current best estimates, these projections need to be viewed with the appropriate uncertainty due to considerable gaps in surveillance of *pfhrp2/3* deletions, as well as heterogeneity in the quality and consistency of previously conducted *pfhrp2/3* surveys^7^. Consequently, the results should be viewed as tools to consider how the two components for mapping the potential spread of deletions—a region’s innate susceptibility for deletions to increase once established (dependent on a region’s malaria transmission intensity, treatment-seeking data and RDT usage data) and the spatial connectivity to regions with high levels of deletions—may interact to drive the spread of deletions. Despite their simplicity, these results could help guide control interventions to stem the threat of *pfhrp2/3* deletions, particularly in identifying regions that need to be prioritized for surveillance to provide accurate data before deciding whether to switch front-line RDTs. Outside regions that have already switched front-line RDTs are countries including Senegal, Zambia and Kenya.

Fewer regions are identified as high risk based on the prospective risk score compared to the innate risk score for two primary reasons. First, the prospective risk score incorporates estimates of the proportion of RDTs in use in a country that are not only HRP2 based. Consequently, countries that primarily use non-HRP2-based RDTs, such as Rwanda (primarily using Pf-RDTs and/or pan-RDTs based on the Global Fund and President’s Malaria Initiative data^25^), will not select for *pfhrp2* deletions. Second, the prospective risk score is seeded with current estimates of *pfhrp2* deletion prevalence in each country. Countries without surveys or <1% *pfhrp2* deletions, such as Tanzania, are predicted to reach the 5% threshold slower than in the innate risk scenario, which explores timelines from a starting frequency of 1% *pfhrp2* deletions. We chose to produce two risk maps (the innate and prospective risks) because robust molecular surveys of *pfhrp2/3* deletions have not been conducted across all regions. Although surveillance for *pfhrp2/3* deletions has increased rapidly since the widespread introduction of RDTs, by the start of 2023 surveys had been conducted in only 22 countries in Africa^3^. For the prospective risk score, we made the simplifying assumption that countries without surveys have 0% *pfhrp2* deletion frequency. If this assumption is incorrect, the prospective risk score will underestimate the risk in these countries.

The innate risk score differs from the prospective risk score by simply focusing on the risk that *pfhrp2* deletions pose once present in a region (and assuming that the region has not switched to non-HRP2-based RDTs alone or in combination with HRP2). Providing both risk scores has several advantages. The innate risk score can be used to confirm that the model correctly identifies regions in which deletions have rapidly increased as high risk. Indeed, the maps of innate risk (Fig. 3) correctly identify the Horn of Africa as a region of consistently high risk. The innate risk score can also be used to address additional questions relevant to malaria policies, including where to prioritize surveillance given plateauing levels of funding and competing demands^1^. For example, if deciding among countries without previous surveys, the innate risk score can be used to identify countries predicted to select for deletions fastest and therefore in greatest need of surveillance and/or early transition to non-HRP2-based RDTs. Last, among countries that have switched away from HRP2-based RDTs, the innate risk score provides an indication of whether these regions would still select for deletions if they switched back to HRP2-based RDTs.

Our approach has several important limitations. First, our exploration of international spread employs a simplistic approach for how deletions are exported between regions. Second, the model parameters carry a high degree of uncertainty. Our estimates of fitness costs are derived from model fitting to a handful of surveys with large differences in the number of samples, which led to a high prediction of the inferred fitness costs, suggesting smaller fitness costs than observed from in vitro experiments^20^. Therefore, they may not reflect the fitness costs associated with *pfhrp2* deletion in parasites outside the Horn of Africa and we do not consider the potential for new *pfhrp2*-deleted strains to emerge with increased fitness. Once additional longitudinal deletion data are available, selection coefficients can be more accurately inferred and fitness costs should be estimated again. However, the degree of uncertainty in certain key parameters, such as malaria prevalence, highlights the need for data to provide more precise estimates of key drivers of *pfhrp2/3* selection. These same data are needed to model the spread of artemisinin partial resistance^26^, which is now spreading in a number of regions in Africa^27–29^. Third, our model assumes that malaria prevalence and treatment will remain constant in the future. Fourth, the country-specific estimates of linkage between *pfhrp2* and *pfhrp3* deletions provided here assume that the dynamics of these two loci are at equilibrium and no selective forces are acting to pull certain genotypes, such as deletion of both *pfhrp2* and *pfhrp3*, to higher levels. However, we have observed a significant relationship between deletions and malaria prevalence that aligns with recent mechanistic explanations of how *pfhrp3* deletions arise and may be driven by low malaria prevalence^30^. If malaria prevalence falls in a region, in addition to the increased selection of *pfhrp2* deletions that occurs at low prevalence, the frequency of *pfhrp3* deletions may also increase, furthering the selection of *pfhrp2* deletions. In response, additional surveillance data of both *pfhrp2* and *pfhrp3* deletions are needed, which can be leveraged to test hypotheses of how non-RDT-mediated processes drive *pfhrp3* deletion emergence and subsequently create a selective niche for *pfhrp2* deletions. Last, although we have modeled how HRP2-based RDTs create a selective pressure for *pfhrp2* deletions, this process does not capture the historic process by which *pfhrp2* deletions have emerged in South America, which occurred without this pressure. These results are, however, still relevant in identifying that these regions are susceptible to selecting for deletions, given the low malaria prevalence if they relied on HRP2-based RDTs, while also noting that a greater understanding of the fundamental biology and evolution that led to the selection of *pfhrp2* deletions in regions in South America is needed.

The issues surrounding spread of *pfhrp2/3* deletions are not unique to malaria. Management strategies for controlling RDT-evasive genotypes can be borrowed from the drug-resistance management literature, which provides evaluations of how multiple antimalarial therapies can be deployed^31,32^. RDTs employing multiple proteins for diagnosis (for example, Pf-HRP2 and Pf-LDH) are analogous to combination therapies, in that a parasite lineage would need to acquire two genetic mechanisms simultaneously to evade detection. Deployment of both HRP2-based RDTs and non-HRP2-based RDTs or microscopy in a single population is similar to the multiple first-line therapy^33^ approach of slowing down resistance, in that an RDT-evasive parasite is likely to undergo diagnosis with a different RDT in the next patient whom it infects. These approaches would first need to be field tested to ensure adequate procurement, distribution and compliance before evaluating their potential for slowing down or reversing the evolution of RDT evasion. Furthermore, these strategies become challenging for areas sympatric for both *P. falciparum and P. vivax*, for which we would need new RDTs at scale to address the current absence of WHO-prequalified combination tests that use Pf-pLDH instead of, or in addition to, HRP2 for *P. falciparum* detection. The decreased sensitivity for LDH relative to HRP2 may, however, still result in a selective advantage, although likely greatly reduced.

In conclusion, this study provides a refined and updated prediction model for the emergence of *pfhrp2/3* deletions. Despite its limitations, our models offer valuable insights that can help policy-makers prioritize surveillance and future deployment of alternative RDTs, leveraging our interactive tool to identify the regions that are consistently identified as high risk. It should also signal to test developers and manufacturers where new markets are likely to emerge first for alternatives to exclusive HRP-RDTs. As our understanding of the complex processes driving *pfhrp2/3* deletions improves and more data become available, we will continue to refine and update our predictions and monitor the increasingly concerning threat posed by *pfhrp2/3* deletions.

## Methods

### *P. falciparum* transmission model

In this study, we employed a previously developed, individually based, mathematical model of *P. falciparum* malaria transmission to simulate the selection of *pfhrp2* deletions^17^. The model monitors the transmission of *pfhrp2*-deleted parasites and wild-type parasites (that is *pfhrp2*^+^) between human and mosquito hosts. We describe the model in brief here (see ref. ^17^ for the full model description and parameters) before detailing further considerations related to *pfhrp3* dynamics and the data sources used to parameterize the model for simulating *pfhrp2* deletions globally.

Individuals are born with maternally acquired immunity that decays within the first 6 months, rendering them susceptible to infection from infectious mosquito bites. Exposure depends on the entomological inoculation rate, which is location specific. The rate at which individuals are bitten by mosquitoes increases with age and is also heterogeneous across the population due to individual-level biting heterogeneity. On infection, individuals acquire either a *pfhrp2*-deleted parasite or a wild-type parasite. This is determined by the genotype frequency of *pfhrp2-*deleted parasites in humans 30 d previously, which accounts for the lags of human exposure, parasite gametocytogenesis and sporozoite development in mosquitoes. After a short latent period, infected individuals either develop clinical symptomatic disease (probability determined by their level of blood-stage immunity, with immunity increasing with age and exposure) or progress as an asymptomatic infection. Symptomatic individuals may seek treatment and they are assumed to be successfully treated unless they are infected with only *pfhrp2*-deleted parasites and the decision to treat is determined only by a positive HRP2-based RDT. All other possible outcomes from an individual seeking treatment (nonadherence to negative RDT outcome, positive HRP2-based RDT due to crossreactivity with HRP3 epitopes, microscopy or alternative RDT (not exclusively reliant on HRP2), used for diagnosis or the individual being treated without being tested) result in the individual being successfully treated. Once treated, individuals undergo a prophylactic period before returning to susceptibility. Asymptomatically infected individuals recover more slowly, with detectability influenced by immunity levels. Superinfection is incorporated, with asymptomatically infected individuals exposed at the same rate as susceptible individuals. Acquired strains from previous infection are naturally cleared after a period similar to the duration of an asymptomatic infection that has not been extended due to superinfection. All infected states are infectious to mosquitoes, with infectivity dependent on detectability (serving as a surrogate for asexual parasite density). Mosquitoes become infected at a rate dependent on human population infectivity and become infectious after approximately 10 d, reflecting the extrinsic incubation period. The model has been parameterized by fitting it to data on the interrelationship of entomological inoculation rate, parasite prevalence, clinical disease incidence and severe disease incidence. The model has also been shown to accurately capture the selection and relationship between *pfhrp2* deletion frequency and transmission intensity in the DRC^17^ and later used to explain seasonal patterns in the detection of *pfhrp2* deletions^34^. Full mathematical details are available in ref. ^17^.

#### *Pfhrp3* dynamics

In a previous modeling analysis, we assumed a fixed probability of 25% that an individual infected with parasites with only *pfhrp2* deleted (that is, *pfhrp3* present) would test positive by HRP2-based RDTs due to crossreactivity with HRP3 epitopes. To more accurately capture the role of *pfhrp3*, we conducted a scoping review of RDT performance on *pfhrp2*^−^*/pfhrp3*^+^ clinical infections to estimate the probability that a positive RDT would occur if *pfhrp3* is present. Second, we noted that *pfhrp3* deletions are frequently found at higher frequencies than *pfhrp2* deletions, despite the latter providing a greater advantage than the former with regard to the ability to evade diagnosis by HRP2-based RDTs^3^. This observation reflects the mechanistic^30^ and soft selective processes that are hypothesized to result in the emergence of *pfhrp3* deletions^5^. This observation is in contrast to the strong selective sweeps associated with *pfhrp2* deletions due to RDT-based test and treatment that cause *pfhrp2* deletions to be selected on both genetic backgrounds, but more strongly on a *pfhrp3*-deleted background^5^. Consequently, we continue to explicitly model only *pfhrp2* deletions in our model and estimate the probability that a *pfhrp2*-deleted parasite has an intact *pfhrp3* gene. If *pfhrp3* is intact, the probability that an individual will yield a positive HRP2-based RDT is determined by the probability of HRP3 crossreacting, which is estimated later as part of a model-fitting exercise. In effect, we model the probability that an individual whose parasites have only *pfhrp2* deletions would have circulating HRP3 due to intact *pfhrp3* and that these yield a positive HRP2-based RDT due to crossreactivity with HRP3 epitopes.

To estimate the association or LD (between genes on different chromosomes) between *pfhrp2* and *pfhrp3* deletions, we used all data uploaded by February 2025 from the WHO Malaria Threat Maps^3^ data to calculate, per study, the total number of *pfhrp2*^−^*/pfhrp3*^−^, *pfhrp2*^−^*/pfhrp3*^+^, *pfhrp2*^+^*/pfhrp3*^−^ and *pfhrp2*^+^*/pfhrp3*^+^ samples. To mitigate against likely differences in assay sensitivity and specificity between surveys, we included in our analysis the surveys that also used an alternative diagnostic (microscopy or non-HRP2-based RDTs) and surveyed symptomatic patients. From the resultant 2 × 2 table, we calculated the normalized coefficient of LD, $D{\prime} \,$, given by:

1 $$D{\prime} =\frac{D}{{D}_{\max }}$$

where $D$ is the coefficient of LD and ${D}_{\max }$ is the theoretical maximum difference between the observed and expected haplotype frequencies, given by:

2 $${D}_{\max }=\left\{\begin{array}{cc}\max \{-{p}_{{\mathrm{A}}}{p}_{{\mathrm{B}}},-(1-{p}_{{\mathrm{A}}})(1-{p}_{{\mathrm{B}}})\} & {\rm{when}}\,D < 0\\ \min \{{p}_{{\mathrm{A}}}(1-{p}_{{\mathrm{B}}}),(1-{p}_{{\mathrm{A}}}){p}_{{\mathrm{B}}}\} & {\rm{when}}\,D > 0\end{array}\right.$$

where *p*_A_ and *p*_B_ are the frequencies of *pfhrp2* and *pfhrp3* deletions, respectively. To estimate the likelihood that *pfhrp2* deletions arise without *pfhrp3* deletions, we calculated the proportion of all *pfhrp2*-deleted infections without *pfhrp3* deletions. For each continent, we fit a beta-binomial distribution (to account for overdispersion across studies) to the calculated study proportions, with the estimated mean used to represent the probability that *pfhrp2* deletions would arise without *pfhrp3* deletions. We also estimated the relationship between the proportion of *pfhrp2* and *pfhrp3* in samples and malaria prevalence (estimated using Malaria Atlas Project data^35^) using overdispersed binomial generalized linear models to describe the observed number of deletions in each survey and the number of samples tested.

### Model parameters for modeling the selection of *pfhrp2* globally

#### Creation of database of model parameters associated with the strength of selection for *pfhrp2/3* gene deletions

Based on previous modeling efforts, we identified a list of risk factors that impact the speed of selection of *pfhrp2* deletions (Extended Data Table 1). We conducted an extensive literature and database review to source estimates for each of the risk factors at the first administrative unit (or national level if not available subnationally) for all countries with stable malaria transmission. We used a three-step process by which we arrived at estimates for each of the risk factors. In overview, in step 1, we undertook a scoping review to identify whether suitable primary databases for each risk factor were available. In step 2, we conducted a literature review to identify additional estimates to supplement or update the databases identified in step 1. In step 3, we assessed whether the estimates from steps 1 and 2 provided additional data or insight beyond those produced by previous mathematical modeling exercises conducted by the Malaria Atlas Project as part of their commodities forecast modeling exercise^21^, before collating data sources to be used as inputs into the *pfhrp2* transmission model (Supplementary Fig. 13). This decision was made because the Malaria Atlas Project commodities database provides estimates of the treatment cascade at a national level (Supplementary Fig. 14), largely produced using statistical models fit with the DHS, Malaria Indicator Surveys, country DHIS2 databases and the WHO World Malaria Report, alongside socioeconomic covariates sourced from the Institute for Health Metrics and Evaluation. Consequently, we sought to identify first whether primary data existed that could be used, before relying on the commodity database estimates if primary data were insufficient.

In step 1, we conducted a scoping review of suitable databases already available for each of the factors, which have been collated by international organizations and entities such as the WHO Global Health Observatory (https://www.who.int/data/gho/data/themes/topics/topic-details/GHO/malaria-testing-diagnosis), the Global Fund’s Price and Quality Reports^25^, the President’s Malaria Initiative RDT distribution data, ACTwatch project publications^36^ and DHS data. The databases identified were standardized at the subnational or national level where appropriate and those identified and how they were standardized for each risk factor are given in Supplementary Table 2.

In step 2, a literature review was conducted to identify additional sources to fill the gaps identified in the databases in step 1 for a number of the risk factors. We constructed relevant search queries for each risk factor (Supplementary Table 3), which were queried against PubMed. Publications were screened based on inclusion and exclusion criteria relevant for each risk factor (Supplementary Table 4), with the full text of identified publications screened and relevant data extracted for each risk factor (Supplementary Table 5). All studies from which data were extracted, as well as the identified target antigen(s) of all RDT listed in volume distribution data, are available in Supplementary Data 1.

Most of the databases that we identified for sourcing parameters in step 1 are the same as those used by the Malaria Atlas Project to inform their commodities forecast modeling exercise. With regard to adherence to RDT diagnostic test outcome, no primary database was identified and the only suitable data source was that estimated by the Malaria Atlas Project. From our literature review, we did not identify any studies since 2015 that passed inclusion criteria. With regard to RDT brand volume data, databases from the Global Fund’s Price and Quality Reports^25^ and the President’s Malaria Initiative^37^ provided RDT volume data for all countries in Africa with malaria except for Equatorial Guinea and Gabon. Although 25 additional studies passed inclusion criteria for the literature search regarding RDT brands, none of the studies included data on Equatorial Guinea and Gabon. In addition, the included studies provided reports on the brands of RDTs used as part of specific scientific investigations and did not necessarily reflect national RDT types used. Last, regarding the size of the private market, we identified 48 studies from 21 countries, of which 4 were from Asia. A range of different measures of the private versus public sector were observed (percentage RDT manufacturer sales to private versus public, surveys of treatment-seeking behavior, analysis of DHS Service Provision Assessment surveys). In addition, DHS data also provide reports on where treatment seeking was sought in a number of DHS survey rounds, which are the underlying data used by the Malaria Atlas Project for modeling test adherence.

Based on the limited primary data, we ultimately relied on the Malaria Atlas Project commodities database for most risk factors, because it provided consistent, nationally representative estimates that were not substantially improved on by additional primary or literature sources. Exceptions to this were made only where data were lacking (for example, RDT brand volumes to inform the proportion of testing that uses HRP2-based RDT). The final inputs for the malaria transmission model and their parameter source are provided in Supplementary Table 6. We accounted for uncertainty in model parameters as follows: for estimates from the Malaria Atlas Project commodity dashboard, the 95% credible intervals estimated during creation of the dashboard^22^ were used for all-cause (private and public) care seeking, microscopy use and test nonadherence. For malaria prevalence, we used the 95% CIs of slide prevalence in 2–10 year olds provided publicly by the Malaria Atlas Project^35^. No uncertainty was available and thus not considered for the proportions of RDT brands used that target only Pf-HRP2.

#### Refining estimates of fitness costs associated with *pfhrp2* deletions

One notable uncertainty for modeling *pfhrp2-*deleted parasites is whether deleted parasites suffer a fitness cost and how that fitness cost impacts the probability of deleted parasites being transmitted onward. Asexual fitness costs have been measured by conducting pairwise competition experiments in vitro, suggesting a fitness cost of 8.7% (relative fitness of 91.3%) for *pfhrp2*-deleted parasite strains and 11.3% (relative fitness of 88.7%) for strains with both *pfhrp2* and *pfhrp3* deletions^20^. These fitness costs were estimated by comparing the growth of *pfhrp2* and/or *pfhrp3* knocked-out strains against a common competitor strain. Consequently, the inferred fitness costs reflect the impact on asexual parasite growth in mixed infections. However, it is unknown whether these measured fitness costs translate to a reduction in onward infection (how we model parasite fitness costs). In addition, previous feeding assay studies have highlighted the importance of measuring the fitness of both asexual and sexual stages to fully characterize the impact on population-level trends^38^.

To estimate the fitness costs associated with *pfhrp2* deletions in our model, we used our transmission model to model the selection of *pfhrp2* deletions in Eritrea and Ethiopia at each first administrative unit. We chose Eritrea and Ethiopia for this parameter estimation exercise because both countries contain at least three surveys collected over time and represent known ‘hot spots’ of *pfhrp2/3* deletions in Africa that have also been shown to cause symptomatic infection. In addition, the surveys include data on *pfhrp3* deletions, which allow for the probability that *pfhrp2* deletions occur with *pfhrp3* deletions to be estimated for each location (revealing that *pfhrp2* deletions were rarely observed without *pfhrp3* deletions). Djibouti was not included because, to date, there have been fewer than three surveys over time among known symptomatic patients.

We statistically compared the modeled frequency of *pfhrp2* deletions against representative *pfhrp2* surveys from the WHO Malaria Threat Maps to jointly infer parameter values for both the comparative fitness costs and the crossreactivity of HRP3 epitopes. We used a Bayesian approach, with a flat prior for the fitness cost, with bounds centered on the fitness cost estimated in the in vitro fitness study^20^ (relative fitness parameter bound between 0.8 and 0.99) and a beta distribution (*α* = 13, *β* = 15) for the probability of HRP3 crossreacting informed. This prior was informed by studies of the performance of HRP2-based RDTs on *pfhrp2*^−^*/pfhrp3*^+^ samples in Ethiopia, which observed 46.2% (12 of 26) of samples yielding a positive RDT^5^. Although other studies in Djibouti^14^ and Uganda^39^ reported lower crossreactivity (0 of 5 and 1 of 10 samples crossreacting, respectively), we chose a prior based on the Ethiopian study, given the location of the *pfhrp2* surveys to which we are fitting in Eritrea and Ethiopia and because no data were available in Eritrea due to previous studies either only observing *pfhrp2*^−^*/pfhrp3*^−^ samples or not testing *pfhrp2*^−^*/pfhrp3*^+^ samples with RDT. The log(likelihood) values were calculated for each study by assuming that the proportion of *pfhrp2* deletions was described by an overdispersed binomial distribution, with the number of samples genotyped in each study used as the number of trials. Median estimates and 95% CIs for each parameter were obtained from 1,000 draws from the posterior parameter space.

### *Pfhrp2* deletion risk scores

In our previous analysis, we created risk scores of ‘HRP2 concern’. To create these scores, we simulated trends in the prevalence of *pfhrp2*-deleted mutants across SSA. These simulations included estimates of the mean, microscopy-based, *P. falciparum* prevalence in 2–10 year olds (*Pf*PR_2–10_) in 2010 by the first administrative unit and estimates of the proportion of cases seeking treatment from previously modeled estimates using the DHS and the Malaria Indicator Cluster Surveys^40^. The time taken for the proportion of infections with all strains *pfhrp2* deleted to reach 20% was recorded and classified to map areas of HRP2 concern under four qualitative classifications. This approach relied, however, on a different metric (namely the proportion of infections with all strains *pfhrp2* deleted) to the 5% false-negative RDTs due to the *pfhrp2* deletion metric subsequently adopted by the WHO for deciding when to switch RDTs^24^. This metric is based on the proportion of clinically relevant infections that would be misdiagnosed due to *pfhrp2/3* gene deletions. To address this discrepancy, we produced maps of two new risk scores—the ‘innate risk’ score and the ‘prospective risk’ score—based on the proportion of clinically relevant infections that would be misdiagnosed due to *pfhrp2/3* gene deletions. To create these new maps, we used updated estimates from 2020 for the parameters described in Extended Data Table 1 and assumed that these estimates remain constant going forward, that is, malaria transmission intensity, treatment-seeking data and RDT usage data remain the same as estimated in 2020.

#### Innate risk score

The first risk score, the innate risk score, is the innate potential for *pfhrp2* deletions to spread once established in a region based solely on the region’s malaria transmission intensity, treatment-seeking data and adherence to diagnostic test outcome. Informed by the current 5% WHO threshold, we defined the innate risk score as the time taken for the percentage of clinical cases to be misdiagnosed by Pf-HRP2-based RDTs to increase from 1% (previously shown to be a suitable threshold for defining establishment of *P. falciparum* genetic traits under positive selection^26^) to 5%. We then used a similar approach to ref. ^17^ to categorize each region’s innate risk score. Here a region’s risk is classified as high, moderate or slight, defined as reaching the 5% threshold within 6, 12 and 20 years, respectively, or marginal risk if 5% is not reached within 20 years. Importantly, we did not incorporate data on the current types of RDT used in that country (these were used in the prospective risk score). Consequently, the innate risk score reflects the risk that deletions would spread in a region if all types of RDTs used were HRP2 based. Although most countries continue to use only HRP2-based RDTs, a number of countries in SSA have switched to non-HRP2-based RDTs: Eritrea, Djibouti and partially Ethiopia. In these countries, the innate risk score thus conveys the risk that is still posed if those countries reverted back to only HRP2-based RDTs.

To estimate the innate risk score for each administrative level 1 region, we first estimated the selection coefficient (the annual percentage change in logit genotype frequency^41^) for clinical cases to be misdiagnosed by Pf-HRP2-based RDTs. We estimated selection coefficients using the following approach: we first created 8,748 unique parameter sets that equally span the range observed globally for six model simulation parameters that capture the drivers of *pfhrp2/3* deletions detailed in Extended Data Table 1: (1) the malaria prevalence; (2) the probability of an individual seeking treatment and being effectively treated after having received a diagnostic test (capturing treatment-seeking rates for fever, proportion of these occurring in the private sector, proportion of individuals seeking care who receive a diagnostic test, the type of RDT used); (3) the adherence to test outcomes for deciding on treatment; (4) the proportion of all diagnoses that occur using microscopy; (5) the relative fitness of *pfhrp2*-deleted parasites; and (6) the probability that an individual infected with only *pfhrp2*-deleted parasites yields a positive HRP2-based RDT due to the parasites not having a *pfhrp3* deletion and the resultant HRP3 crossreacting with the RDT, yielding a positive test.

For all parameter combinations, 5 stochastic realizations of 100,000 individuals were simulated for 40 years to reach equilibrium first before simulating the selection of *pfhrp2* deletions over the next 20 years, with a starting frequency of *pfhrp2* deletions of 6%. The 6% was chosen based on recommendations made by a previous modeling study^41^, which recommends selecting an allele frequency as low as possible to reflect the condition under which most selection occurs, but also high enough to reduce stochastic noise in allele spread and allow for more accurate estimation of selection coefficients from modeling outputs. From each simulation, we recorded the monthly proportion of clinically relevant infections that would be misdiagnosed due to *pfhrp2/3* gene deletions (that is, clinical infections only infected with *pfhrp2* deletions and not yielding a positive test due to HRP3). We subsequently calculated selection coefficients (the annual percentage change in proportion of misdiagnosed clinical cases) for each simulation repetition by linear regression of the log(odds) of a clinical case being misdiagnosed (Supplementary Fig. 15)^42,43^.

We next trained an ensemble machine learning model (for full details, see ‘Ensemble machine learning model for predicting selection coefficients’) to predict selection coefficients based on model simulation parameters detailed in Extended Data Table 1. This approach provides a statistical model that replicates the underlying transmission model behavior that can be subsequently generalized to any transmission setting. From these models, we predicted how quickly the 5% threshold will be reached once *pfhrp2* deletions have been established in a region (defined as 1% frequency based on previous antimalarial resistance modeling exercises^26^). Uncertainty in selection coefficients due to stochastic variation in model simulations was also estimated using a similar statistical modeling framework.

#### Prospective risk score

The innate risk score, while capturing the underlying selection dynamics, does not incorporate data on the current distribution of *pfhrp2/3* deletions in Africa. The second risk score, which we called the prospective risk score, is calculated from a prospective modeling approach designed to explore different scenarios of how *pfhrp2* deletions may continue to spread in Africa, based on current estimates of the prevalence of *pfhrp2* deletions from the WHO Malaria Threat Maps. Although there are considerable uncertainties in the prevalence of gene deletions across Africa^7^ and identifying the true denominator in reported surveys is challenging^3^, these estimates represent our best understanding of the current genotype frequency of *pfhrp2* deletions in Africa. In countries without molecular surveillance data, we assumed the current frequency of *pfhrp2* deletions to be 0%.

Given the difficulty in estimating the rate at which malaria parasites under selection spread geographically^44^, we used a simple model of parasite movement to describe how *pfhrp2/3* deletions spread between the first administrative units. To simulate the spread between regions, we made the simplifying assumption that *pfhrp2* deletions are exported from an admin level 1 region once *pfhrp2* deletions have been found in 25% of clinical cases; when this threshold has been reached, *pfhrp2*-deleted parasites are seeded into neighboring regions such that neighboring regions reach 1% genotype frequency after 1 year. Once a region reaches a 1% genotype frequency, the future trajectory of deletions in that region is solely determined by the selection coefficient estimated for the region for a given parameter set. Given the use of a single fixed selection coefficient for each region, this assumes that malaria prevalence and case management in each region remain constant over time. Using this approach, we simulated a range of possible timelines for *pfhrp2* deletions in Africa.

#### Ensemble machine learning model for predicting selection coefficients

From our simulations previously described, we produced a dataset of selection coefficients calculated using simulation outputs corresponding to 5 stochastic realizations for each of the 8,748 unique sets of the 6 model simulation parameters that capture the drivers detailed in Extended Data Table 1. We used the generated dataset to train an ensemble statistical model to predict selection coefficients based on these six parameters described. Of the simulated datasets 25% were held back as an out-of-sample dataset to be used for evaluating the performance of the trained statistical models and to test for overfitting. The remaining 75% of the simulated data was used for training 3 different statistical models (shape-constrained additive models, bagged multivariate regression splines and Bayesian regularized neural networks) to predict selection coefficients using the six varied transmission model parameters. Statistical model performance was evaluated based on the root mean-squared error (RMSE). Optimum model-fitting hyperparameters based on RMSE were first identified by scanning over hyperparameters for each model before fitting each model. When identifying hyperparameters and training the final model, *K*-fold crossvalidation sets were produced by splitting the training data into 20 sets of training data with the results of the crossvalidation subsequently averaged to reduce any bias from the crossvalidation set chosen. We calculated the performance of each trained model by calculating the RMSE for each model when tested using the holdout dataset. To construct our final ensemble model, we simply calculated the average across the three models, weighted by their RMSE from the holdout test.

Uncertainty in selection coefficients due to stochastic variation in model simulations was also estimated using a similar statistical modeling framework (75% data split, hyperparameter tuning and 20-fold crossvalidation). For each parameter set, we used each trained model to first predict the selection coefficient. Next, we calculated the absolute prediction error by comparing the model prediction against the selection coefficient for each stochastic realization, before calculating the s.d. in the error across stochastic realizations. We trained a Bayesian regularized neural network model to predict the s.d. in error before calculating robust CIs given by ±1.96 × s.d.

**Extended Data Fig. 1:**
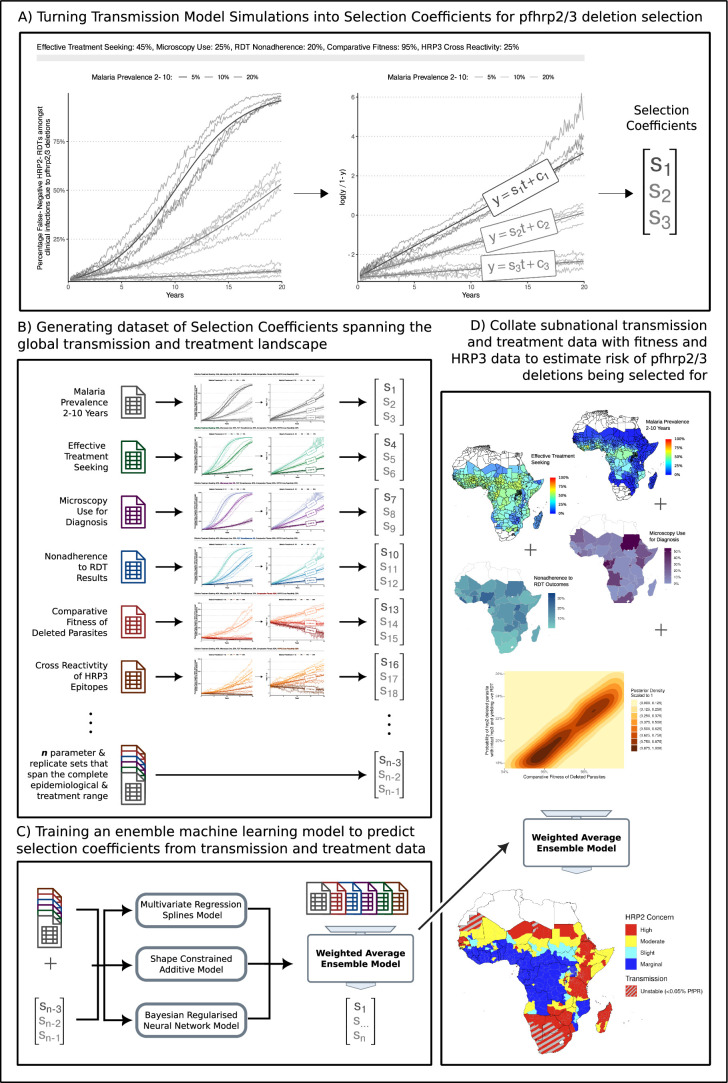
Workflow schematic for modelling and mapping the global risk of selection and spread of *pfhrp2/3* deletions. **a**) Estimation of selection coefficients from stochastic model simulations. **b**) Generating training data using simulation studies that feed into **c**) a statistical model for estimating selection coefficients. **d**) The final model is used to estimate the risk posed by *pfhrp2/3* deletions using collated subnational covariates and parameters from model fitting exercises.

We used the weighted average ensemble model to predict selection coefficients for each first administrative unit based on the malaria prevalence and treatment-related data for the administrative unit. A complete schematic of this modeling pipeline is given in Extended Data Fig. 1.

### Ethics and inclusion

This study exclusively utilized publicly available, anonymized datasets, with no primary data collection involving humans or animals. The deletion data analyzed were obtained from the WHO Malaria Threat Maps, reflecting extensive international collaboration and inclusivity. We gratefully acknowledge the diverse contributions of these global research communities. All co-authors actively participated from the early stages of project design through to data interpretation and manuscript preparation. Local and regional researchers responsible for generating these publicly accessible datasets were appropriately cited and acknowledged throughout. Authorship reflects significant contributions across project conceptualization, funding acquisition, data analysis and interpretation, manuscript drafting and critical revisions.

### Reporting summary

Further information on research design is available in the Nature Portfolio Reporting Summary linked to this article.

## Online content

Any methods, additional references, Nature Portfolio reporting summaries, source data, extended data, supplementary information, acknowledgements, peer review information; details of author contributions and competing interests; and statements of data and code availability are available at 10.1038/s41591-025-03974-3.

## Supplementary information

Supplementary Information Supplementary Figs. 1–15 and Tables 1–6.

Reporting Summary

Supplementary Video 1 Prospective risk modeling time series animation of the spread of clinically relevant infections misdiagnosed due to *pfhrp2/3* gene deletions in Africa based on prospective risk score modeling method.

Supplementary Data Literature review findings literature review included studies and the identified target antigen(s) of all RDTs listed in volume distribution data in which are available.

## Data Availability

The data for the R Shiny tool at https://worldhealthorg.shinyapps.io/DeletionRiskExplorer are available via GitHub at https://github.com/rjzupkoii/WHO-Malaria-pfhrp23 (v.0.1.2). All data used in and generated by this analysis are available in a reproducible and version-controlled R research compendium via GitHub at https://github.com/OJWatson/hrpup (v.0.3.0)^45^. This includes external datasets: WHO Malaria Threat Maps (https://apps.who.int/malaria/maps/threats)^3^. No access restrictions apply; the repository and all associated data will remain publicly available indefinitely.

## Extended data

**Extended Data Table 1:** Drivers of *pfhrp2/3* deletion selection included in mathematical modelling

Drivers of *pfhrp2/3* selection	Impact on speed of selection for *pfhrp2/3* deletions	Data sources used
Malaria Prevalence	Lower malaria prevalence will increase selection pressure by increasing the probability that individuals are only infected with *pfhrp2/**3* deleted parasites and thus more likely to not be treated. Additionally, lower malaria prevalence will increase the probability that an infected individual will develop a symptomatic infection (due to lower immunity at lower transmission intersities), which in turn influences the infected individual’s likelihood to seek treatment.	Malaria Atlas Project maps of slide positivity ages 2–10^35^.
Microscopy-based diagnosis	The use of microscopy for malaria diagnosis will decrease selection pressure by negating the selective advantage conferred by *pfhrp2/3* deletions.	WHO World Malaria Report ‘proportion of cases confirmed by diagnostic’ table, with missing data imputed using all other collected model parameters.
Treatment-seeking rate for fever	Increased treatment-seeking will increase the rate at which the selective advantage conferred by *pfhrp2/3* is able to be realised by evading diagnosis and treatment.	Commodities Forecast Dashboard by the Malaria Atlas Project^21^, which uses Demographic and Health Surveys (DHS), Malaria Indicator Surveys (MIS), Multiple Indicator Cluster Surveys (MICS) and AIDS Indicator Surveys (AIS) in generalized additive mixed model (GAMM) to predict treatment seeking patterns over time.
Proportion of treatment-seeking for fever in the private sector.	Low use of malaria rapid diagnostic tests has been shown to exist in the private market in a number of locations^46^. If the use of RDTs is lower in the private market than in the public sector then selection pressure will decrease with an increasingly large private drug market.	DHS/MIS Surveys used in GAMM for estimating treatment seeking from any (medical) source and for estimating treatment seeking in the public sector.
Proportion of individuals seeking care who receive diagnostic test	Low use of any diagnostic test for guiding treatment decisions will reduce selection pressure for *pfhrp2/3* deletions.	DHS data (surveys in Africa asking if care-seeking febrile children received a finger/heel prick).
Nonadherence to RDT outcomes	Nonadherence to RDT outcomes (treating RDT negative individuals) will decrease selection pressure by negating the selective advantage conferred by *pfhrp2/3* deletions.	Commodities Forecast Dashboard by the Malaria Atlas Project^21^, which uses a statistical model of the probability of care-seeking fevers receiving any antimalarial informed by DHS and MIS data.
RDT brands	The use of non-HRP2-based RDTs will negate the selective advantage conferred by *pfhrp2/3* deletions.	Global Fund Price and Quality Reporting and President’s Malaria Initiative data on volumes of RDT test types and brands used.
Cross-reactivity of PfHRP3 epitopes	Increasing cross-reactivity between PfHRP3 epitopes and PfHRP2-based RDTs will decrease selection pressure for *pfhrp2* deletions.	Estimate based upon WHO Malaria Threat Maps data and studies reporting performance of HRP2-based RDTs on *pfhrp2-/pfhrp3* + (^5,14,39^).
Fitness costs associated with *pfhrp2/3 gene* deletions.	Fitness costs associated with *pfhrp2/3* gene deletions will reduce the transmissibility of gene deleted parasites.	Parameterised via model fitting to Eritrean and Ethiopian *pfhrp2/3* deletion data, with priors from *in vitro* competition assay data^20^.

Each row corresponds to a distinct driver, with the theoretical or observed impact of each driver explained. The final column describes the different data sources and methodologies used to estimate values for each driver that are used as parameters in this modelling study. Parameter values for each driver were sourced at the national level, except for malaria prevalence and treatment-seeking rates, which were sourced at the first administrative unit. See Supplementary Information for full methodology.

## Code availability

All software developed, software packages used and code used for data analysis are available in a reproducible and version-controlled R research compendium via GitHub at https://github.com/OJWatson/hrpup (v.0.3.0)^45^. Source code is available via GitHub at https://github.com/rjzupkoii/WHO-Malaria-pfhrp23.
