# Supplementary material for: Global risk of selection and spread of Plasmodium falciparum histidine-rich protein 2 and 3 gene deletions
Source: Nat Med. 2025 Oct 6;31(10):3372–9. doi: 10.1038/s41591-025-03974-3 (PMC12532711; doi:10.1038/s41591-025-03974-3)
Supplement: Supplementary file 1 — Supplementary Figs. 1–15 and Tables 1–6. [file 41591_2025_3974_MOESM1_ESM.pdf]

nature medicine

Supplementary information https://doi.org/10.1038/s41591-025-03974-3

Global risk of selection and spread of *Plasmodium falciparum* histidine-rich protein 2 and 3 gene deletions

In the format provided by the authors and unedited

Supplementary Figures

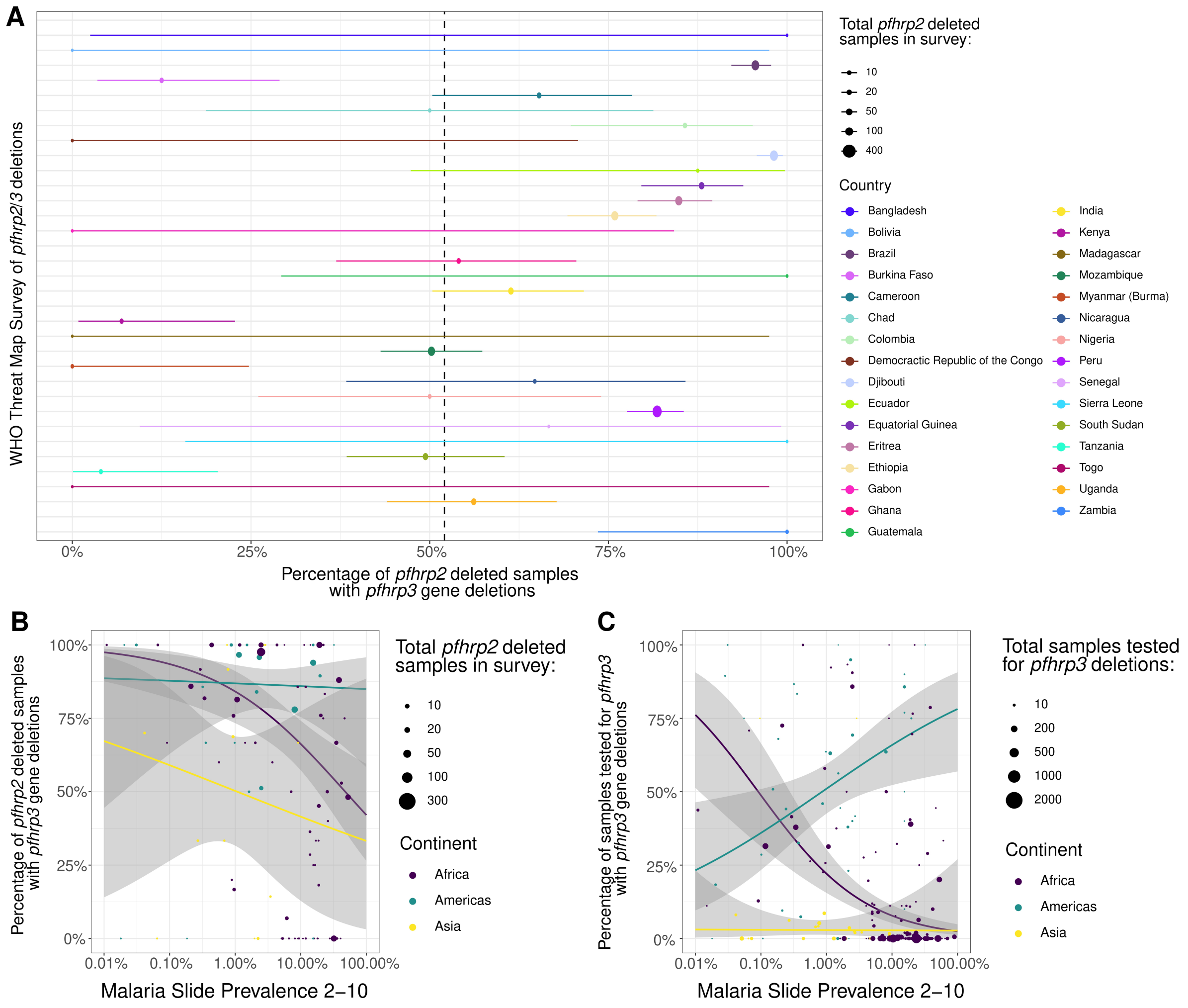

Supplementary Figure 1. Distribution and independence of *pfhrp2/3* deletions globally collated in the WHO Threat Maps database. A) Percentage of *pfhrp2*-deleted samples also with *pfhrp3* deletions by survey. The mean and 95% confidence interval is shown with points and ranges. Sample size per country: Australia (19), Burkina Faso (32), Bangladesh (1), Bolivia (1), Brazil (248), Cameroon (49), Democratic Republic of the Congo (3), Colombia (35), Germany (1), Djibouti (272), Ecuador (8), Eritrea (198), Ethiopia (195), Gabon (2), United Kingdom (4), Ghana (37), Equatorial Guinea (92), Guatemala (3), India (88), Ireland (1), Kenya (29), Madagascar (1), Myanmar (Burma) (13), Mozambique (201), Nigeria (18), Nicaragua (17), Peru (385), Senegal (3), Sierra Leone (2), South Sudan (85), Chad (10), Togo (1), Tanzania (25), Uganda (73), Zambia (12). B). Relationship between the percentage of *pfhrp2*-deleted samples also with *pfhrp3* deletions against malaria slide prevalence in 2-10 year-olds based on Malaria Atlas Project estimates. C). Relationship between the percentage of samples with *pfhrp3* deletions and malaria prevalence. In all plots, the point size represents the number of samples from each survey used to derive estimates. In both B) and C) Binomial regression model fit (blue) shows the mean relationship, with the 95% confidence interval of the regression fit shown with shaded bands. The regression relationships are shown for each continent.

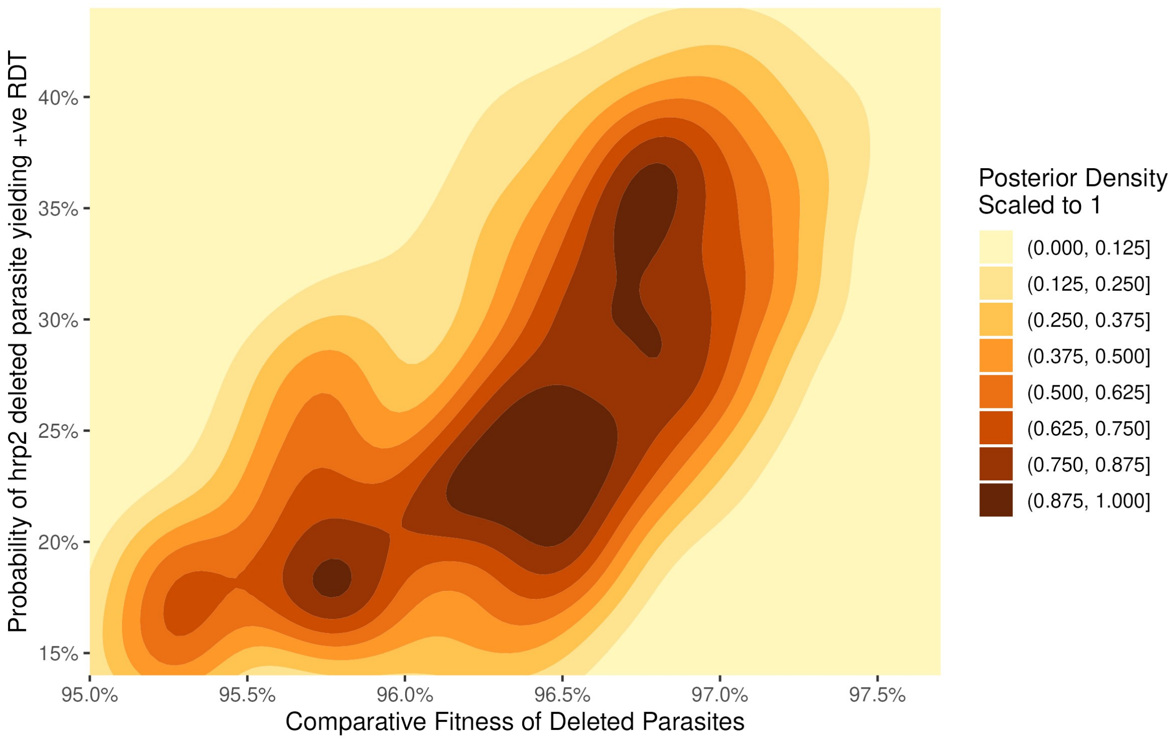

Supplementary Figure 2. Parameter estimation of fitness and HRP3 cross-reactivity. Selection trends for studies in Ethiopia and Eritrea reporting frequencies of *pfhrp2* deletions were created based on historical malaria prevalence and treatment seeking trends and spanning likely ranges for HRP3 cross reactivity and comparative fitness costs. 1000 parameter pairs were drawn from the posterior distribution and the resultant posterior density scaled to a maximum of 1 is shown, with darker colours showing a higher area of likelihood.

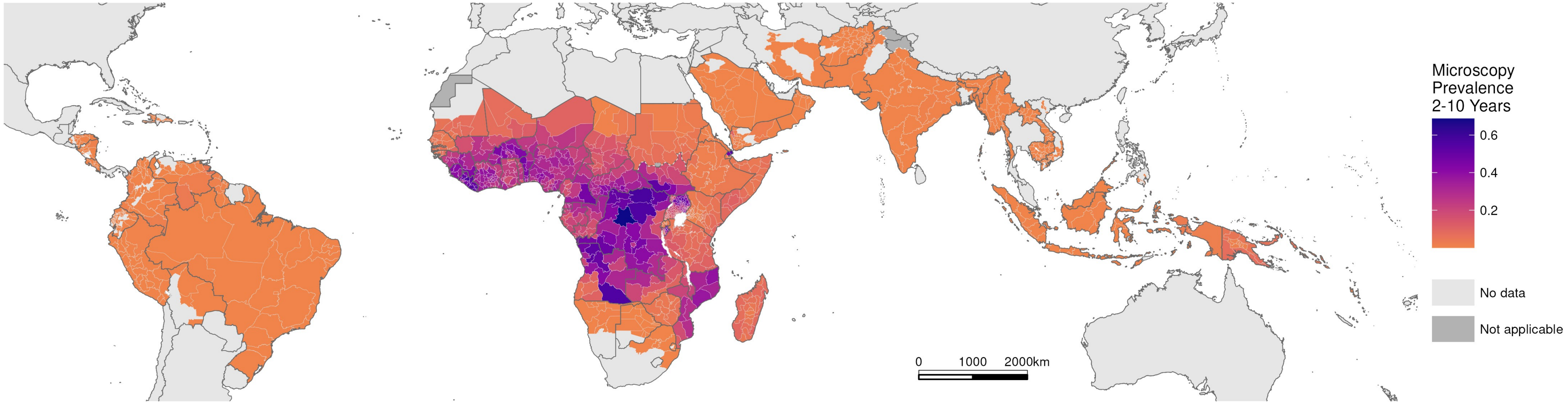

Supplementary Figure 3. Median malaria slide prevalence in children aged 2-10 in 2020 based on Malaria Atlas Project estimates and aggregated to the first administrative unit.

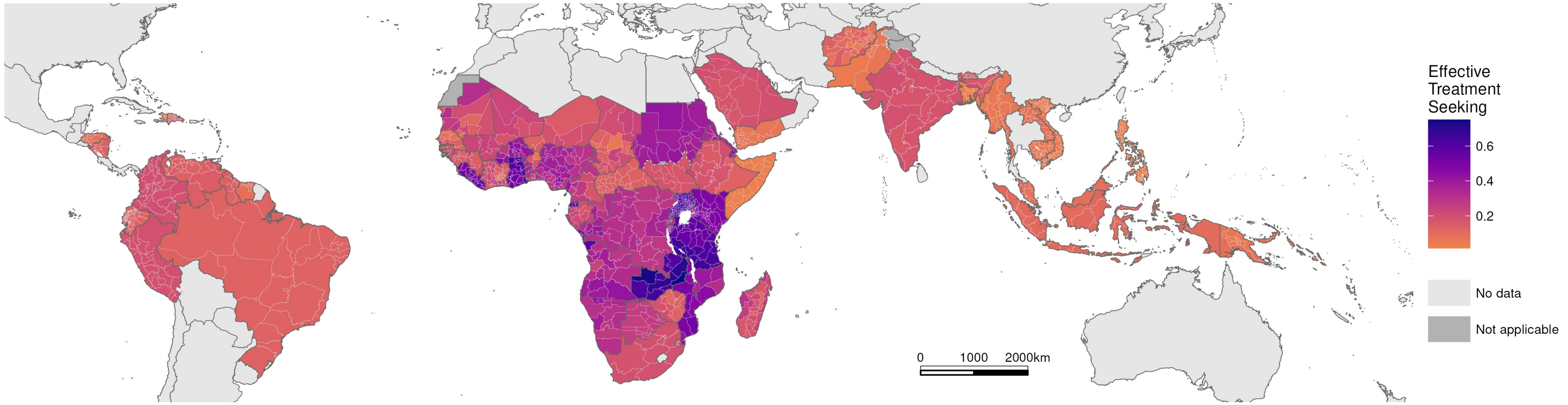

Supplementary Figure 4. Median effective treatment in 2020. Effective treatment seeking reflects the probability that an individual with symptomatic malaria seeks treatment and is subsequently treated. Estimates are shown at the first administrative unit.

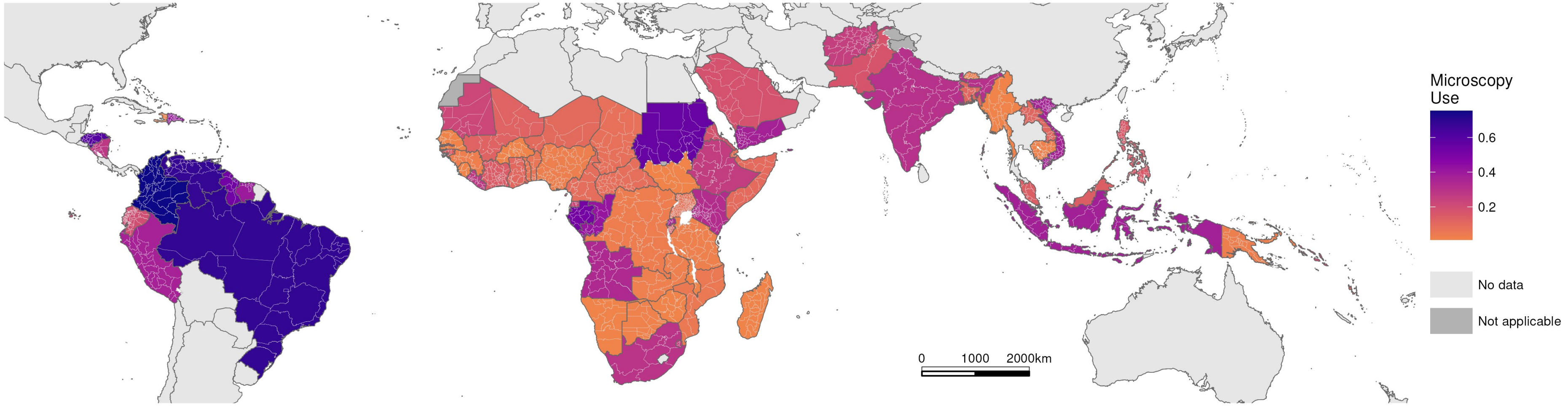

Supplementary Figure 5. Proportion of all diagnostic testing conducted using microscopy in 2020. Estimates available at the national level.

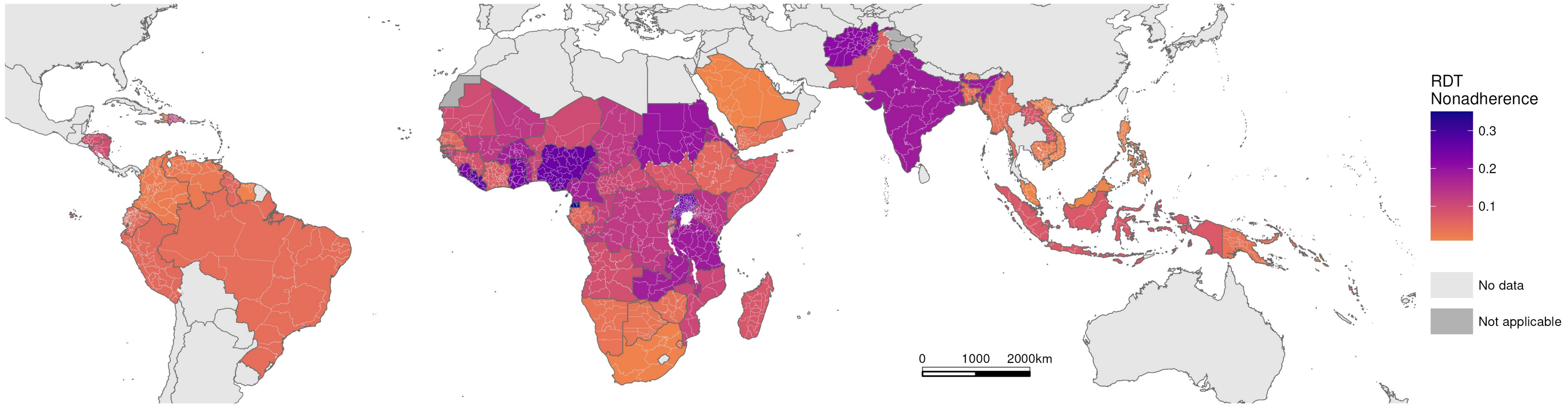

Supplementary Figure 6. Proportion of false-negative RDT tests that are subsequently treated for malaria, i.e. non-adherence to RDT test outcomes in 2020. Estimates available at the national level.

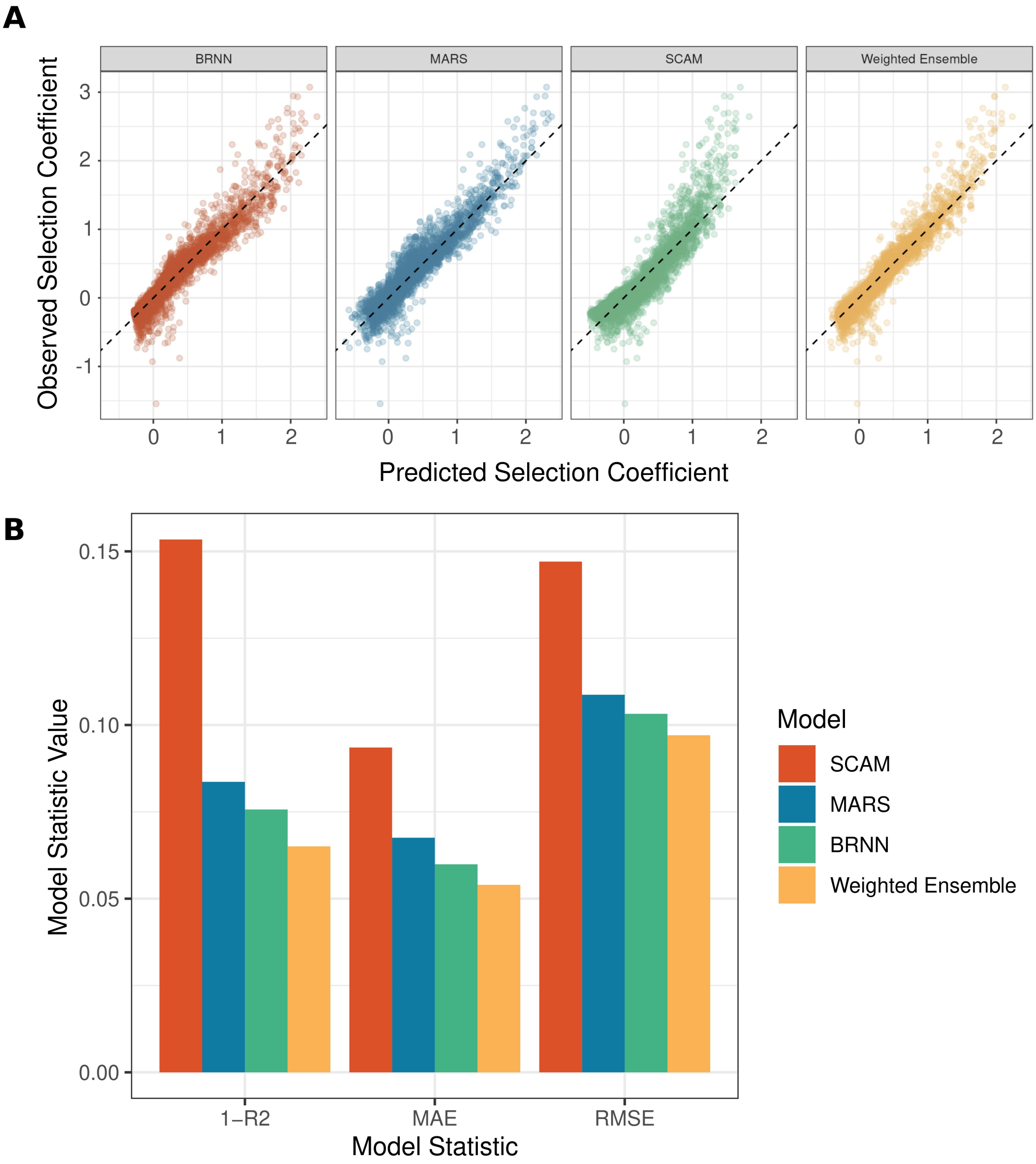

Supplementary Figure 7. Ensemble model fitting performance on hold-out test data. A) Model prediction selection coefficients are shown against the observed selection coefficients, with y = x trend line shown with a dashed line. B) Model performance summary statistics for each individual model alongside the weighted ensemble model.

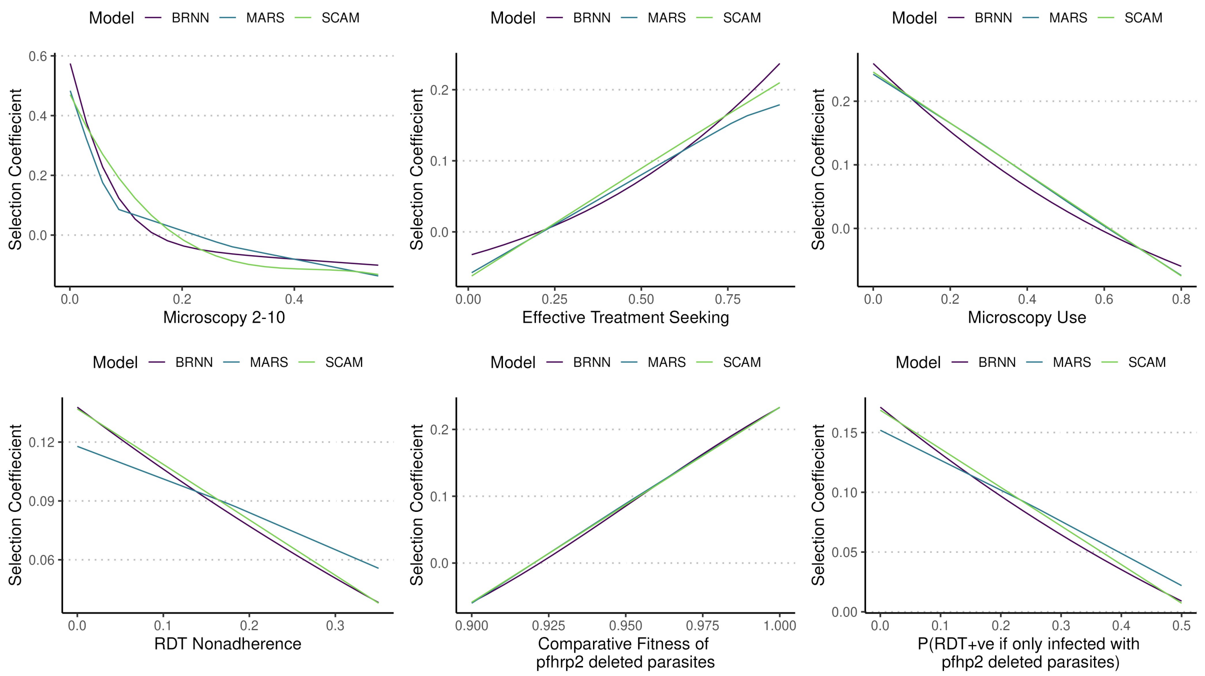

Supplementary Figure 8. Partial dependence plots of each individual model used in the ensemble model for predicting selection coefficients. Partial dependence is shown across the six model inputs that were explored when modelling the risk of *pfhrp2* deletions. Models used were: BRNN - Bayesian Regularised Neural Network, MARS - Multivariate Adaptive Regression Splines, SCAM - Shape Constrained Additive Models

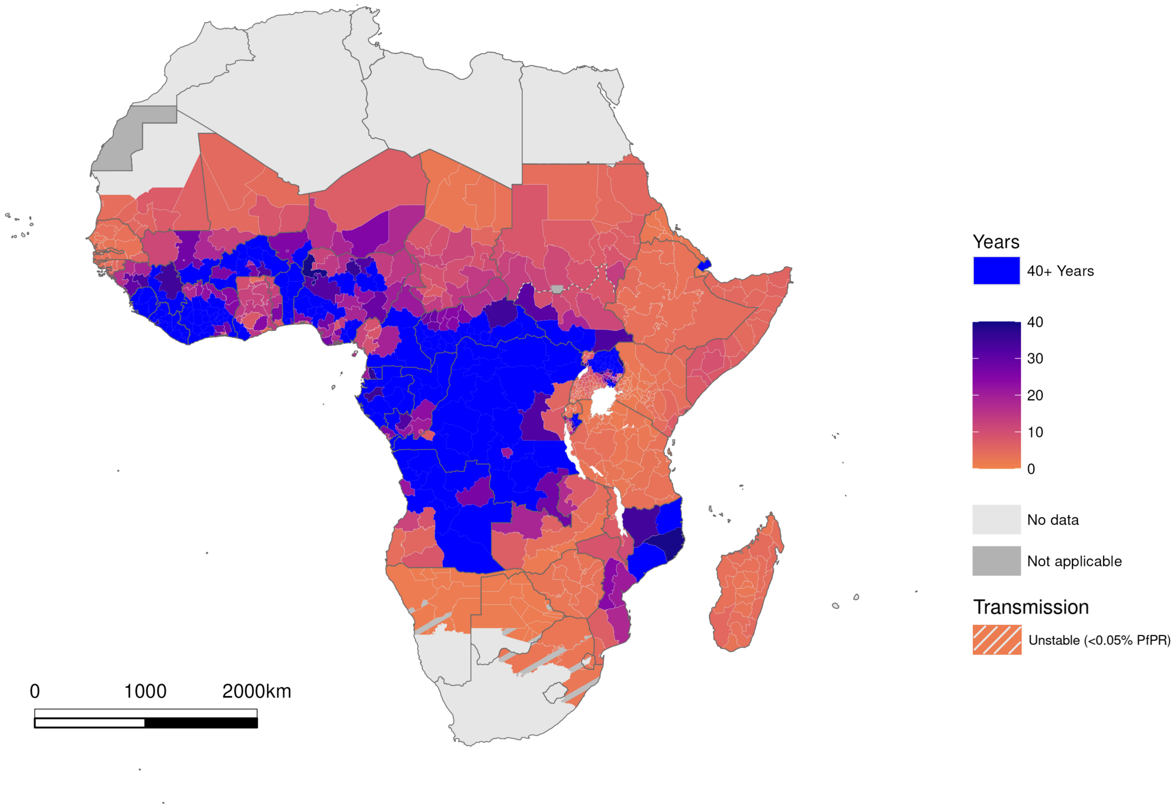

Supplementary Figure 9. Predicted central estimates for the time in years for the percentage of clinically relevant infections misdiagnosed due to *pfhrp2/3* gene deletions to increase from 1% to 5% in Africa. Regions estimated to not reach 5% within 40 years are shown in blue. Regions with very low, unstable malaria transmission (defined as <0.05% malaria prevalence) are shown with diagonal grey lines.

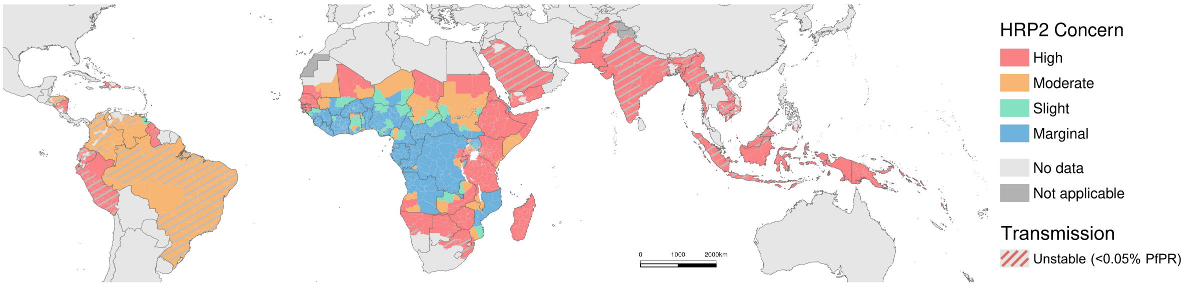

Supplementary Figure 10. Central estimates of the Innate risk score for the concern caused by *pfhrp2* deletions globally. High (red), moderate (yellow) and slight (teal) risk represent >5% of clinically relevant infections misdiagnosed due to *pfhrp2/3* gene deletions in less than 6, 12 and 20 years respectively, and marginal risk (blue) represents <5% in 20 years. Regions with very low, unstable malaria transmission (defined as <0.05% malaria prevalence) are shown with diagonal grey lines.

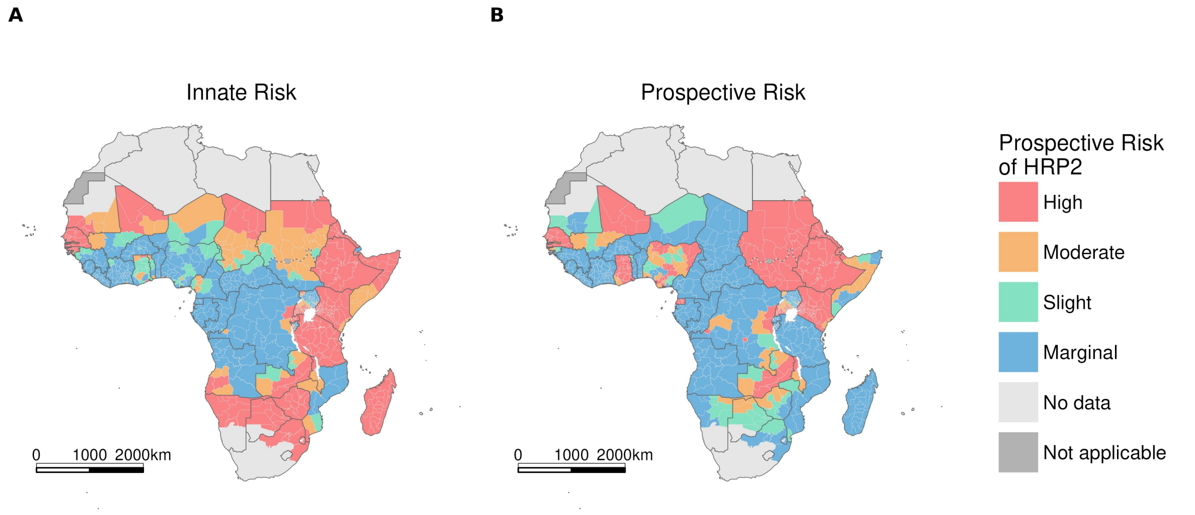

Supplementary Figure 11. Innate and Prospective risk scores for Africa. Risk scores are presented for our central estimates of covariates. High (red), moderate (yellow) and slight (teal) risk represent >5% of clinically relevant infections misdiagnosed due to *pfhrp2/3* gene deletions in less than 6, 12 and 20 years respectively, and marginal risk (blue) represents <5% in 20 years.

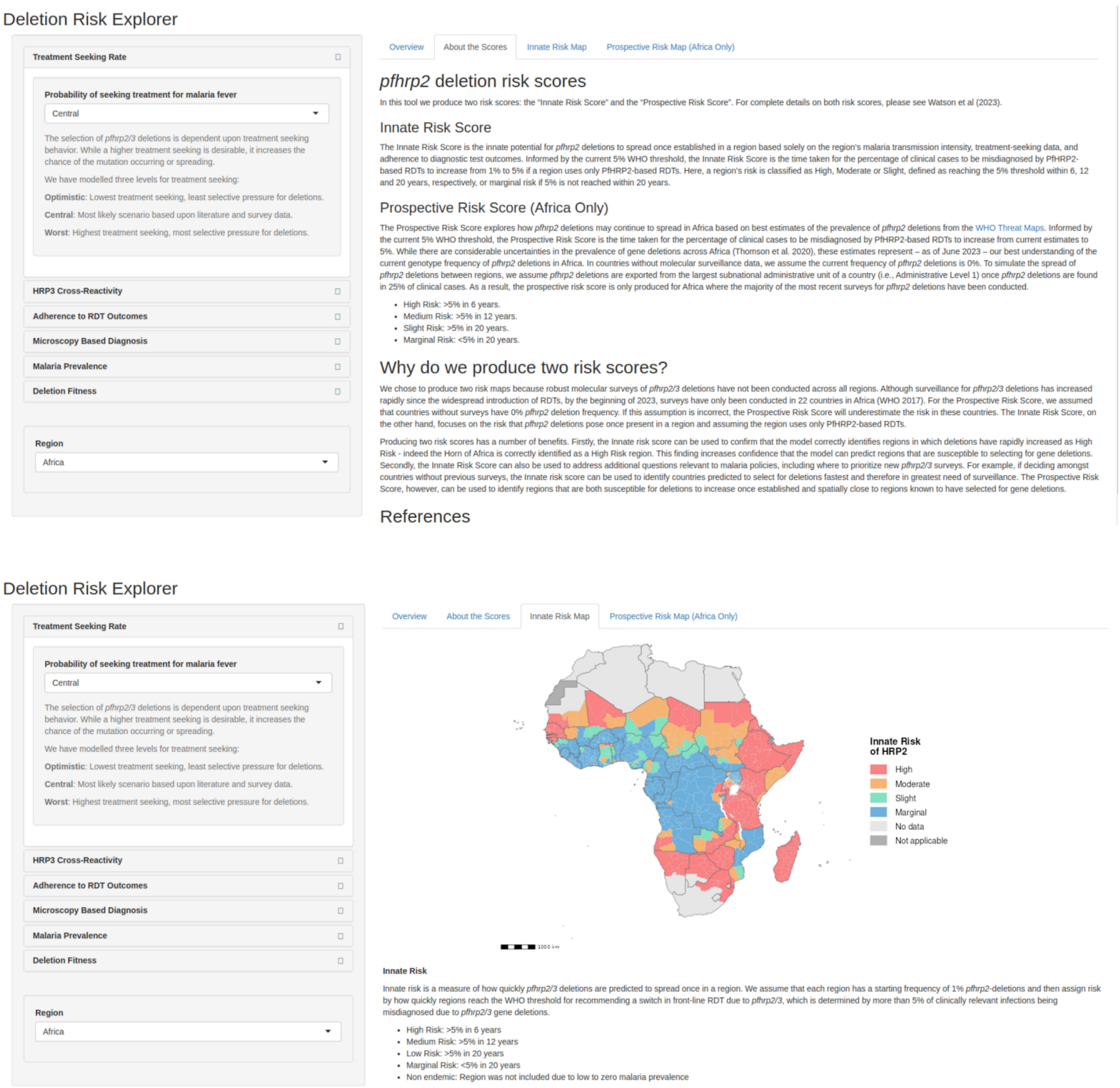

Supplementary Figure 12. Example interactive interface for exploring the full range of uncertainty in *pfhrp2* risk. The interface allows users to explore how changing scenarios explored (Worst, Central, Optimistic case scenarios with respect to *pfhrp2* deletions spread) impacts the risk posed by *pfhrp2* deletions.

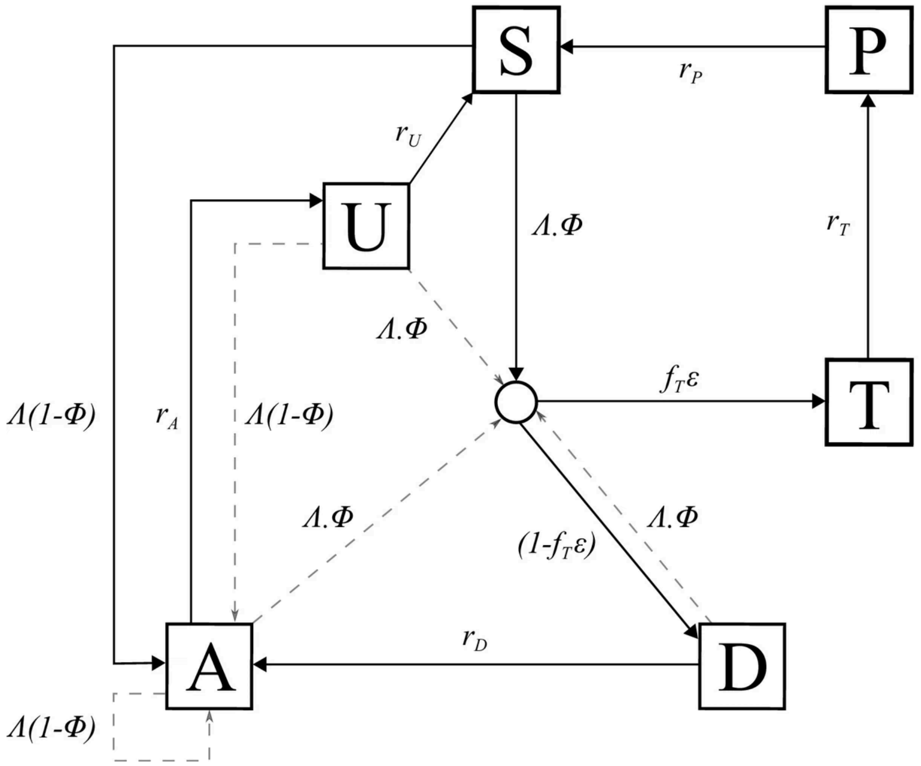

Supplementary Figure 13. Flow diagram for the human component of the transmission model, with dashed arrows indicating superinfection. S, susceptible; T, treated clinical disease; D, untreated clinical disease; P, prophylaxis; A, asymptomatic patent infection; U, asymptomatic sub-patent infection; r_x, rates of transition between compartments; f_T, effective treatment seeking (seeking treatment that will result in effective treatment unless diagnostic test results in a false-negative outcome due to *pfhrp2* deletions; Λ, force of infection; Φ, probability of developing clinical symptoms that may trigger seeking treatment; ε, probability of HRP3 epitopes present and cross reacting to yield positive HRP2-based RDT.

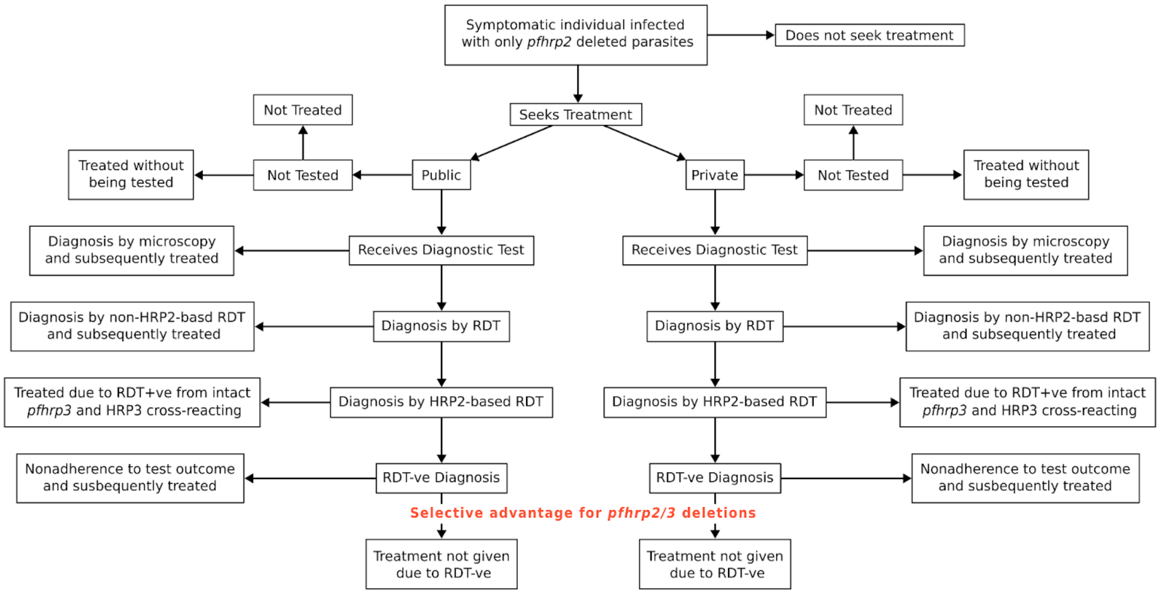

Supplementary Figure 14. Full treatment cascade pathways. Routes to treatment outcomes are aggregated when included in the transmission model, with parameters for each process determined from our parameter and literature review.

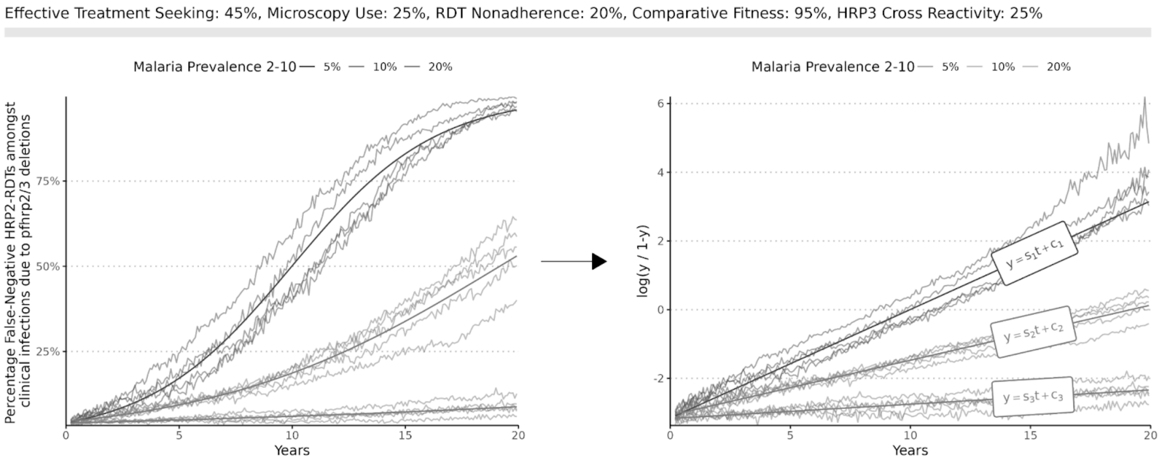

Supplementary Figure 15. Conversion from model simulations to selection coefficients. For a given parameter set (effective treatment-seeking: 45%, microscopy use: 25%, RDT nonadherence: 20%, comparative fitness: 95%, HRP3 cross reactivity: 25%), the simulated percentage of false-negative HRP2-based RDTs amongst clinical infections due to *pfhrp2/3* deletions (y) is converted to log odds (y/1-y), with the gradient calculated to estimate the selection coefficient.

Supplementary Tables

Supplementary Table 1. Binomial modelling of the impact of malaria prevalence on *pfhrp2/3* status globally

	***pfhrp3* gene deletion prevalence amongst *pfhrp2* deleted samples (95% CI)**	***pfhrp3* gene deletion prevalence (95% CI)**
**Malaria Prevalence**	-0.3017 (-0.3340, -0.2695)	-0.5444 (-0.5698, -0.5190)
	p: <2e-16	p: <2e-16
**Observations**	243	260
**Log Likelihood**	-3,428.49	-5,038.33

Supplementary Table 2. Primary data sources for *pfhrp2* risk factors identified and methods used to collate these into national or subnational estimates

Factor	Parameter	Data Base Identified	Method
#1	Proportion of microscopy versus *hrp2*-based rapid diagnostic test (RDT)	WHO Global Health Observatory (Feb 2023)	Retrieved country-level data on suspected malaria cases (#suspects), cases examined by microscopy (#microscopy), and cases examined by RDT (#rdt).<br><br>Microscopy share of testing calculated as:<br><br>$proportion\ of\ microscopy = \frac{\#microscopy}{\#microscopy + \#rdt}$
#2	Proportion of *hrp2*-based RDTs among all RDTs used	Global Fund Price and Quality Reports (PQR); President Malaria Initiative RDT distribution data; WHO Public Reports for In Vitro Diagnostics	Extracted country-level RDT volumes by brand. Records without country or brand were removed. Antigen targets cross-checked with WHO In Vitro Diagnostics reports. Proportion of HRP2-only RDTs estimated from three most recent years of data.
#3	Proportion of children with fever who sought treatment advice	Demographic and Health Surveys (DHS)	National and subnational estimates of % of febrile under-5 children seeking any care/treatment extracted from most recent DHS surveys.
#4	Proportion of suspected malaria cases who received any diagnostic test	WHO Global Health Observatory (Feb 2023)	Retrieved country-level data on suspected malaria cases (#suspects), cases examined by microscopy (#microscopy), and cases examined by RDT (#rdt).<br><br>Testing coverage calculated as:<br><br>$testing\ coverage = \frac{\#microsscopy + \#rdt}{\#suspects}$
#5	Malaria prevalence in children 2-10 years old	Demographic and Health Surveys (DHS); Malaria Indicator Surveys (MIS)	Estimates of malaria prevalence in children 2–10 years extracted from DHS/MIS surveys.
#6	Proportion of RDT-negative suspected cases that did not receive any antimalarials (adherence to RDT results)	No primary database available	Malaria Atlas Project Commodity Forecasting tool includes modelled estimates of the probability that RDT-negative individuals still receive antimalarials.
#7	Antimalarial market share of private versus public sector	ACTwatch publications	Extracted published country-level data on market share split between public and private sectors.

Supplementary Table 3. PubMed search queries and categories used to identify literature sources for each risk factor

Category	PubMed search terms
Publication Year (X to Y)	(X:Y[pdat])
Malaria	("Malaria"[Mesh] OR malaria*[tiab] OR Plasmodium*[tiab] OR "malaria, falciparum"[MeSH Terms])
Fever	("Fever/diagnosis"[Mesh] OR "Fever/epidemiology"[Mesh] OR "Fever/therapy"[Mesh])
Diagnostic test	("diagnostic tests, routine"[MeSH Terms] OR "Malaria/diagnosis"[MAJR] OR ("diagnostic"[All Fields] AND "tests"[All Fields] AND "routine"[All Fields]) OR "routine diagnostic tests"[All Fields] OR ("diagnostic"[All Fields] AND "test"[All Fields]) OR "diagnostic test"[All Fields])
Microscopy	("microscopies"[All Fields] OR "microscopy"[MeSH Terms] OR "microscopy"[All Fields])
RDT brand	(("First Response"[All Fields] OR "CareStart"[All Fields] OR "CareStart Malaria"[All Fields] OR "Bioline"[All Fields] OR "Bioline Malaria"[All Fields] OR "SD Bioline"[All Fields] OR "Paracheck"[All Fields] OR "Paracheck Pf"[All Fields] OR "ParaHIT"[All Fields] OR "ICT Malaria"[All Fields] OR "Advy"[All Fields] OR "STANDARD Q"[All Fields] OR "One Step"[All Fields] OR "Abbott"[All Fields] OR "NanoSign"[All Fields] OR "Malaria Ag"[All Fields] OR "Standard Diagnostics"[All Fields] OR "Onsite Pf/Pv"[All Fields] OR "FirstSign -"[All Fields] OR "OptiMAL-IT"[All Fields] OR "Clearview Malaria"[All Fields] OR "Malaria P.f/Pan"[All Fields] OR "SD BIOLINE"[All Fields] OR "Biocredit"[All Fields] OR "Hexagon Malaria"[All Fields] OR "ParaHIT- f"[All Fields] OR "Parahit f"[All Fields] OR "Parahit-f"[All Fields] OR "Onsite Pf"[All Fields] OR "Bionote"[All Fields] OR "ICT Diagnostics"[All Fields] OR "Paramax-3"[All Fields] OR "Parascreen"[All Fields] OR "Meriscreen"[All Fields] OR "Falcivax"[All Fields] OR "BinaxNOW Malaria"[All Fields] OR "Loopamp Malaria"[All Fields] OR "Humasis Malaria"[All Fields]) AND "rapid"[All Fields])
Treatment	("Malaria/drug therapy"[Mesh] OR "Antimalarials"[Mesh] OR "artemisinins"[MeSH Terms])
Private sector	("private sector"[MeSH Terms] OR ("private"[All Fields] AND "sector"[All Fields]) OR "private sector"[All Fields] OR "pharmacies"[MeSH Terms] OR pharmacy[All Fields] OR pharmacies[All Fields])
Public sector	("public sector"[MeSH Terms] OR ("public"[All Fields] AND "sector"[All Fields]) OR "public sector"[All Fields])
Pfhrp2/3 deletion	("pfhrp*"[All Fields] AND ("deletable"[All Fields] OR "deletant"[All Fields] OR "deletants"[All Fields] OR "delete"[All Fields] OR "deleted"[All Fields] OR "deleter"[All Fields] OR "deleters"[All Fields] OR "deletes"[All Fields] OR "deleting"[All Fields] OR "deletion s"[All Fields] OR "deletional"[All Fields] OR "gene deletion"[MeSH Terms] OR ("gene"[All Fields] AND "deletion"[All Fields]) OR "gene deletion"[All Fields] OR "deletions"[All Fields] OR "sequence deletion"[MeSH Terms] OR ("sequence"[All Fields] AND "deletion"[All Fields]) OR "sequence deletion"[All Fields] OR "deletion"[All Fields]))
Treatment seeking behaviour	("patient acceptance of health care"[MeSH Terms] OR ("patient"[All Fields] AND "acceptance"[All Fields] AND "health"[All Fields] AND "care"[All Fields]) OR "patient acceptance of health care"[All Fields] OR ("treatment"[All Fields] AND "seeking"[All Fields] AND "behavior"[All Fields]) OR ("treatment"[All Fields] AND "seeking"[All Fields] AND "behaviour"[All Fields]) OR "treatment seeking behavior"[All Fields] OR "treatment seeking behaviour"[All Fields])
Case management	("case management"[MeSH Terms] OR ("case"[All Fields] AND "management"[All Fields]) OR "case management"[All Fields] OR "population surveillance"[MeSH Terms] OR ("therapy"[Subheading] OR "disease management"[MeSH Terms]))
Adherence	("Guideline Adherence"[Mesh] OR "adhere"[All Fields] OR "adhered"[All Fields] OR "adherence"[All Fields] OR "adherences"[All Fields] OR "adherent"[All Fields] OR "adherents"[All Fields] OR "adherer"[All Fields] OR "adherers"[All Fields] OR "adheres"[All Fields] OR "adhering"[All Fields] OR "guideline adherence"[MeSH])
Compliance	("compliances"[All Fields] OR "patient compliance"[MeSH Terms] OR ("patient"[All Fields] AND "compliance"[All Fields]) OR "patient compliance"[All Fields] OR "compliance"[All Fields] OR "compliance"[MeSH Terms])
Health knowledge and practice	("health knowledge, attitudes, practice"[MeSH Terms])

Supplementary Table 4. Inclusion and exclusion criteria for identified publications within each risk factor category

Factor	Inclusion criteria	Exclusion criteria
#1	● Report the number of samples screened by either RDTs or microscopy<br>● Report on public sector<br>● Published from 2021 to 2023<br>● Are in English	● Do not report the number of samples screened by either RDTs or microscopy<br>● Do not report on public sector<br>● Published before 2021<br>● Are not in English
#2	● Report the RDT brand used to diagnose P.falciparum infection<br>● Conduct in the following countries: Afghanistan, Angola, Burkina Faso, Bangladesh, Cote d'Ivoire, Cameroon, Congo, Democratic Republic of Congo, Comoros, Cabo Verde, Ethiopia, Ghana, Gambia, Guinea-Bissau, Guatemala, Guyana, Honduras, Haiti, Indonesia, Laos, Mali, Mozambique, Malawi, Niger, Nigeria, Nicaragua, Nepal, Pakistan, Philippines, Papua New Guinea, Democratic People's Republic of Korea, Zanzibar, Senegal, Solomon Islands, Sierra Leone, Somalia, South Sudan, Sao Tome and Principe, Eswatini, Chad, Togo, Tanzania, Zimbabwe, Burundi, Benin, Bolivia, Bhutan, Djibouti, Eritrea, Guinea, Kenya, Madagascar, Myanmar, Mauritania, Sudan, Timor-Leste, Uganda, Venezuela, Zambia<br>● Report sample collection year<br>● Published from 2020 to 2023<br>● Are in English	● Do not report the RDT brand used to diagnose P.falciparum infection<br>● Do not conduct in the following countries: Afghanistan, Angola, Burkina Faso, Bangladesh, Cote d'Ivoire, Cameroon, Congo, Democratic Republic of Congo, Comoros, Cabo Verde, Ethiopia, Ghana, Gambia, Guinea-Bissau, Guatemala, Guyana, Honduras, Haiti, Indonesia, Laos, Mali, Mozambique, Malawi, Niger, Nigeria, Nicaragua, Nepal, Pakistan, Philippines, Papua New Guinea, Democratic People's Republic of Korea, Zanzibar, Senegal, Solomon Islands, Sierra Leone, Somalia, South Sudan, Sao Tome and Principe, Eswatini, Chad, Togo, Tanzania, Zimbabwe, Burundi, Benin, Bolivia, Bhutan, Djibouti, Eritrea, Guinea, Kenya, Madagascar, Myanmar, Mauritania, Sudan, Timor-Leste, Uganda, Venezuela, Zambia<br>● Do not report sample collection year<br>● Published before 2020<br>● Are not in English
#3	● Report the number/percentage of febrile children who sought care at public facilities<br>● Are not part of DHS program<br>● Published from 2010 to 2023<br>● Are in English	● Do not report the number/percentage of febrile children who sought care at public facilities<br>● Are part of DHS program<br>● Published before 2010<br>● Are not in English
#4	● Report the number of samples screened by either RDTs or microscopy<br>● Report on public sector<br>● Published from 2021 to 2023<br>● Are in English	● Do not report the number of samples screened by either RDTs or microscopy<br>● Do not report on public sector<br>● Published before 2021<br>● Are not in English
#6	● Report the number of cases tested with RDT<br>● Report the number of RDT negative<br>● Report the number of RDT-negative cases who were (not) given antimalarials<br>● Published from 2015 to 2023<br>● Are in English	● Do not report the number of cases tested with RDT<br>● Do not report the number of RDT negative<br>● Do not report the number of RDT-negative cases who were (not) given antimalarials<br>● Published from before 2015<br>● Are not in English
#7	● Report the number private facilities<br>● Report the number public facilities<br>● Published from 2010 to 2023<br>● Are not part of ACTWatch Project<br>● Are in English	● Do not report the number private facilities<br>● Do not report the number public facilities<br>● Published before 2010<br>● Are part of ACTWatch Project<br>● Are not in English

Supplementary Table 4. Search structures, publication yield, and extracted data for each risk factor

Factor	Search structure	Publications Identified	Extracted
#1	<publication from 2021 to 2023> AND <malaria> AND (<diagnostic test> OR <microscopy>) AND <public sector>	16	4
#2	("Afghanistan"[Mesh] OR "Angola"[Mesh] OR "Burkina Faso"[Mesh] OR "Bangladesh"[Mesh] OR "Cote d'Ivoire"[Mesh] OR "Cameroon"[Mesh] OR "Congo"[Mesh] OR "Democratic Republic of the Congo"[Mesh] OR "Comoros"[Mesh] OR "Cabo Verde"[Mesh] OR "Ethiopia"[Mesh] OR "Ghana"[Mesh] OR "Gambia"[Mesh] OR "Guinea-Bissau"[Mesh] OR "Guatemala"[Mesh] OR "Guyana"[Mesh] OR "Honduras"[Mesh] OR "Haiti"[Mesh] OR "Indonesia"[Mesh] OR "Laos"[Mesh] OR "Mali"[Mesh] OR "Mozambique"[Mesh] OR "Malawi"[Mesh] OR "Niger"[Mesh] OR "Nigeria"[Mesh] OR "Nicaragua"[Mesh] OR "Nepal"[Mesh] OR "Pakistan"[Mesh] OR "Philippines"[Mesh] OR "Papua New Guinea"[Mesh] OR "Democratic People's Republic of Korea"[Mesh] OR "Zanzibar"[All Fields] OR "Senegal"[Mesh] OR "Melanesia"[MeSH Terms] OR “Solomon Islands”[All Fields] OR "Sierra Leone"[Mesh] OR "Somalia"[Mesh] OR "South Sudan"[Mesh] OR "Sao Tome and Principe"[Mesh] OR "Eswatini"[Mesh] OR “Swaziland”[All Fields] OR "Chad"[Mesh] OR "Togo"[Mesh] OR "Tanzania"[Mesh] OR "Zimbabwe"[Mesh] OR “Burundi"[Mesh] OR "Benin"[Mesh] OR "Bolivia"[Mesh] OR "Bhutan"[Mesh] OR "Djibouti"[Mesh] OR "Eritrea"[Mesh] OR "Guinea"[Mesh] OR "Kenya"[Mesh] OR "Madagascar"[Mesh] OR "Myanmar"[Mesh] OR "Mauritania"[Mesh] OR "Sudan"[Mesh] OR "Timor-Leste"[Mesh] OR "Uganda"[Mesh] OR "Venezuela"[Mesh] OR "Zambia”[Mesh]) AND <publication from 2020 to 2023> AND <malaria> AND <diagnostic test> AND <RDT brand>	31	25
#3	<publication from 2010 to 2023> AND (<malaria> OR <fever>) AND ("patient acceptance of health care"[MeSH Terms] OR ("patient"[All Fields] AND "acceptance"[All Fields] AND "health"[All Fields] AND "care"[All Fields]) OR "patient acceptance of health care"[All Fields] OR ("treatment"[All Fields] AND "seeking"[All Fields] AND "behavior"[All Fields]) OR ("treatment"[All Fields] AND "seeking"[All Fields] AND "behaviour"[All Fields]) OR "treatment seeking behavior"[All Fields] OR "treatment seeking behaviour"[All Fields]) AND <public sector>	63	0
#4	<publication from 2021 to 2023> AND <malaria> AND (<diagnostic test> OR <microscopy>) AND <public sector>	16	4
#6	<publication from 2010 to 2023> AND <malaria> AND <diagnostic test> AND "rapid"[All Fields] AND “negative”[All Fields] AND <treatment> AND (<case management> OR <adherence> OR <compliance> OR <health knowledge and practice>) NOT ("glucosephosphate dehydrogenase"[MeSH]) NOT ("cost-benefit analysis"[MeSH])	158	0
#7	<publication from 2010 to 2023> AND <malaria> AND <public sector> AND <private sector> AND (<diagnostic test> OR <treatment>)	111	48

Supplementary Table 6. Final data sources used for each risk factor and the methods used to collate these sources to be used as into the *pfhrp2* transmission model. MAP: Malaria Atlas Project, DHS

Parameter	Source(s)	Methods
**Malaria prevalence**	Malaria Atlas Project slide prevalence (ages 2–10)	We extracted the national and subnational median and 95% confidence intervals for slide-prevalence in children aged 2–10 years from the Malaria Atlas Project database for 2020. These estimates are generated by spatial statistical models fitted to household survey data and routine surveillance sources, as described in the MAP methodology.
**Effective treatment seeking**	Malaria Atlas Project Commodity Dashboard (national treatment cascade data);<br><br>Demographic and Health Surveys (subnational care-seeking)	We used Malaria Atlas Project Commodity Dashboard estimates of effective treatment seeking at national level, combining public and private sector cascade components (care-seeking, testing, treatment after positive test, treatment after no test).<br><br>To capture subnational variation, we fit a gradient boosted tree model to under-5 care-seeking data sourced from the Demographic and Health Surveys. Predictors included Malaria Atlas Project healthcare travel time and friction maps, WorldPop population estimates aggregated to the first-administrative region, and national-level socio-economic covariates (health spending, wealth, democracy, urbanisation, inequality) collated from the Economist excess mortality modelling dataset:<br><br>github.com/TheEconomist/covid-19-the-economist-global-excess-deaths-model<br><br>The model was trained with 20-fold cross-validation (75% train, 25% test split) and tuned hyperparameters (learning rate = 0.05, max depth = 12, subsample row ratio = 0.85, subsample column ratio = 0.85).<br><br>Predictions were generated for countries lacking Demographic and Health Surveys data, and rescaled so that the population-weighted national mean matched the Malaria Atlas Project Commodity Dashboard estimate.
**Microscopy use for diagnosis**	Malaria Atlas Project Commodity Dashboard (proportion of RDTs)	We took the Malaria Atlas Project Commodity Dashboard estimates of the proportion of diagnostic tests conducted with RDTs. Microscopy use was derived as the complement (1 – RDT proportion). Following evidence on sectoral practice, we assumed microscopy is concentrated in the public sector only.
**Non-adherence to diagnostic test outcomes**	Malaria Atlas Project Commodity Dashboard (probability of treatment after negative test in private/public sector)	We extracted Malaria Atlas Project modelled probabilities of treatment despite a negative diagnostic test, separately for public and private sectors. These were combined as a weighted average across sectors to obtain the overall probability of non-adherence to diagnostic outcomes.
**Proportion of PfHRP2-only RDTs**	Global Fund Price and Quality Reporting;<br><br>President's Malaria Initiative RDT distribution data	We collated country-level RDT brand volume data from the Global Fund's Price and Quality Reporting system and President's Malaria Initiative RDT distribution data for 2018-2021. Each RDT brand was classified by target antigen, and the country-level proportion of PfHRP2-only RDTs was calculated as the share of distributed test volumes across all brands and years combined.
